# Modulation of microRNome by Human Cytomegalovirus and Human Herpesvirus 6 Infection in Human Dermal Fibroblasts: Possible Significance in the Induction of Fibrosis in Systemic Sclerosis

**DOI:** 10.3390/cells10051060

**Published:** 2021-04-29

**Authors:** Irene Soffritti, Maria D’Accolti, Gloria Ravegnini, Maria-Cristina Arcangeletti, Clara Maccari, Flora De Conto, Adriana Calderaro, Elisabetta Caselli

**Affiliations:** 1 Department of Chemical, Pharmaceutical and Agricultural Sciences and LTTA, University of Ferrara, 44121 Ferrara, Italy; irene.soffritti@unife.it (I.S.); maria.daccolti@unife.it (M.D.); 2 Department of Pharmacy and Biotechnology, University of Bologna, 40126 Bologna, Italy; gloria.ravegnini2@unibo.it; 3 Department of Medicine and Surgery, University of Parma, 43126 Parma, Italy; mariacristina.arcangeletti@unipr.it (M.-C.A.); clara.maccari@unipr.it (C.M.); flora.deconto@unipr.it (F.D.C.); adriana.calderaro@unipr.it (A.C.)

**Keywords:** microRNA, miRNome, human cytomegalovirus, human herpesvirus 6, systemic sclerosis, fibrosis

## Abstract

Human cytomegalovirus (HCMV) and Human herpesvirus 6 (HHV-6) have been reportedly suggested as triggers of the onset and/or progression of systemic sclerosis (SSc), a severe autoimmune disorder characterized by multi-organ fibrosis. The etiology and pathogenesis of SSc are still largely unknown but virological and immunological observations support a role for these beta-herpesviruses, and we recently observed a direct impact of HCMV and HHV-6 infection on the expression of cell factors associated with fibrosis at the cell level. Since miRNA expression has been found profoundly deregulated at the tissue level, here we aimed to investigate the impact on cell microRNome (miRNome) of HCMV and HHV-6 infection in in vitro infected primary human dermal fibroblasts, which represent one of the main SSc target cells. The analysis, performed by Taqman arrays detecting and quantifying 754 microRNAs (miRNAs), showed that both herpesviruses significantly modulated miRNA expression in infected cells, with evident early and late effects and deep modulation (>10 fold) of >40 miRNAs at each time post infection, including those previously recognized for their key function in fibrosis. The correlation between these in vitro results with in vivo observations is strongly suggestive of a role of HCMV and/or HHV-6 in the multistep pathogenesis of fibrosis in SSc and in the induction of fibrosis-signaling pathways finally leading to tissue fibrosis. The identification of specific miRNAs may open the way to their use as biomarkers for SSc diagnosis, assessment of disease progression and possible antifibrotic therapies.

## 1. Introduction

Human diseases associated with tissue fibrosis represent a worldwide concern, due on one hand to the high number of affected individuals and on the other hand to the still not elucidated mechanisms of the fibrosis process [1]. Moreover, the presence of several heterogeneous manifestations and potential etiological agents, together with the lack of validated markers, render very difficult the therapeutic approach. Fibrotic diseases include diverse pathological conditions, such as systemic sclerosis (SSc), nephrogenic fibrosis, sclerodermatous disease, and many other organ-specific disorders mostly impairing the cardiac, kidney, lung, and liver functions. Although such differences, all fibrotic diseases are characterized by accumulation of fibrotic tissue and yet elusive mechanisms [1]. As mentioned above, fibrosis is one of the main features of SSc. This is an autoimmune connective tissue disease of still uncertain etiology characterized by the deposition of excessive extracellular matrix, leading initially to cutaneous thickening and hardening, followed by a gradual and progressive multiorgan involvement, often lethal in the end [2,3,4]. The systemic effects include vasculopathy and immune-dysregulation, which leads to fibrosis and apoptosis of the involved tissues and internal organs, including lungs [5], gastrointestinal tract [6,7], heart [8] and kidneys [9]. The severity of the disease is variable and patients can be classified into limited cutaneous (lcSSc) or diffuse cutaneous (dcSSc) subgroups, based on higher extent of skin involvement [10]. In particular, patients with diffuse cutaneous involvement appear at high risk for quick progression toward internal organs fibrosis, which strikes the lungs, heart, gut, kidneys and ligaments, with high mortality rate. By contrast, the subjects with limited cutaneous involvement (lcSSc) generally present vasculopathy but often have a more favorable outcome.

There is still a lack of certainty about the etiology and pathogenesis of the disease, although there is consensus in that it results from a multifactorial and multistep process, including hereditary genetic predisposition, oxidative stress and environmental factors [11,12,13]. Among this last group of factors, virus infections have been suggested as environmental triggers, with particular focus on those sustained by human *Betaherpesvirinae* subfamily members of *Herpesviridae* virus family, including human cytomegalovirus (HCMV) and human herpesvirus 6 (HHV-6) [14,15,16,17,18,19]. HCMV and HHV-6 are ubiquitous, highly prevalent in the human population, causing asymptomatic or mild infections in the immunocompetent host but severe diseases in the immunocompromised subject; both viruses have been hypothesized to be relevant cofactors in several diseases [20,21]. As a common feature of all herpesviruses, they persist in a latent state in the host after the primary infection, and can reactivate in the susceptible immunocompromised subject. Both viruses are able to infect in vitro and in vivo fibroblasts and endothelial cells (ECs), which represent the main target cells in SSc disease [22,23,24,25,26].

Clues of a possible HCMV involvement in SSc include the detection of HCMV transcripts in ECs from the skin of SSc patients [26], the presence of significantly higher levels of anti-HCMV antibodies in SSc subjects compared to controls [27,28,29,30], the possible role of the above antibodies in recognizing fibroblasts and EC surface antigens, evoking molecular mimicry [31,32], and the presence of specific anti-HCMV CD8+ T cells in SSc patients, associated with the disease severity [15].

As to HHV-6, including the two species HHV-6A and HHV-6B with high genome homology but diverse cell tropism and pathological associations [21,33], the virus was detected in ECs in vivo [34], and in vitro infection of ECs infection was shown to induce production of pro-inflammatory cytokines and impairment of ECs angiogenic properties [35]. HHV-6 has been associated with several autoimmune diseases including connective tissue diseases [17,18,19,36,37,38,39] and was detected frequently in Hashimoto’s thyroiditis patients, a disease often preceding the development of SSc [40]. Recently, HHV-6 was found in the skin of SSc patients and SSc subjects have higher titers of antibodies directed against the HHV-6 U94 protein [16].

Finally, in vitro infection of primary human dermal fibroblasts with HCMV and HHV-6 induced a prompt and sustained expression of pro-fibrotic factors, suggesting a role of these viruses in the progression of tissue fibrosis [14].

Vasculopathy and fibro-proliferative processes are two crucial hallmarks of SSc disease, however the pathogenic connections between such pathways in SSc is poorly understood. Recent studies have focused on exploring the impact of epigenetic factors in order to clarify the molecular mechanisms involved in SSc disease [41,42]. In particular, great attention has been paid to microRNAs (miRNA or miR), short sequences (20–23 nucleotides) of non-coding RNA, which play important roles in regulating the expression of protein coding genes at the post-transcriptional level. The role of miRNAs has been explored in many pathological mechanisms including cancer, fibrosis and autoimmunity, providing potential therapeutic approaches and promising prognostic/diagnostic biomarkers [43]. With regard to SSc pathways, several miRNAs have been implicated in both vascular and fibrotic alterations that characterize the disease [44].

Low levels of miR-152 in SSc dermal microvascular ECs (MVECs) have been linked to endothelial nitric oxide synthase (eNOS) pathway dysfunction and consequent defect in nitric oxide production, condition promoting early SSc vasculopathy [45]. MVECs stimulated with serum from SSc patients evidenced dysregulation of miRNAs related to Toll-like receptor (TLR) signaling, transforming growth factor-beta (TGF-β) pathway activation and Wingless/Int-1 (WNT) signaling, a group of signal transduction pathways associated with endothelial dysfunction [45]. In particular: miR-146b, miR-130b, miR-21, miR-31 and miR-34a were found up-regulated in the skin of SSc patients compared to controls, whereas miR-145 resulted instead down-regulated [46]. Several studies have detected higher levels of miR-155 in the serum and skin of SSc patients, which appears to have a role in both vascular alterations and tissues fibrosis [47,48,49]. Down-regulated levels of miR-194b, implicated in plasminogen activation, have been found in skin biopsies and fibroblasts of SSc patients [50]. miR-130b resulted overexpressed in SSc skin biopsies and was shown to have a regulatory role on endothelin-1 production [51]. Several miRNAs are known to be involved in the regulation of endothelial to mesenchymal transition (EndoMT) [52,53,54], interacting with TGF-β, WNT and NOTCH signaling [55,56,57,58].

Several studies suggested a contribution of miRNAs in the regulation of SSc fibrosis pathway. In particular, miR-29 family include key regulators of collagen expression and fibrosis-associated transcriptional factors. MiR-29 has been detected constitutively decreased in SSc fibroblast and skin [59,60], and was associated with modulation of collagen expression in dermal fibroblasts from SSc patients [61]. The expression levels of several other collagen-related miRNAs, including miR-196a, miR-129-5p and let-7a, appeared downregulated in SSc patients and may contribute to the pathological increased expression of α1 (1) and α2 (2) collagen [62,63,64]. Resistance to apoptosis has been suggested as another possible mechanisms promoting fibrosis in SSc disease. The expression of survivin, a key anti-apoptotic protein, has been linked to apoptosis resistance and increased auto-reactivity during autoimmune diseases [65], and has been found overexpressed in fibroblasts from SSc patients [66]. Alteration of survivin-targeting microRNA, including miR-542-3p, miR-708-5p, miR-125b, has recently been observed in SSc patients and linked to apoptosis activation [67]. In addition, recent data evidenced a down-regulation of miR-125b levels in SSc skin and fibroblast [68], and an altered expression of miR-150-5p, miR-16-5p, and miR-485-5p was found in peripheral blood mononuclear cells (PBMCs) of SSc patients, which negatively correlated with survivin mRNA expression [69].

Notably, several evidences prove that herpesvirus infection may cause significant changes in cellular miRNA expression, and these alterations may play a role in triggering or promoting pathogenic pathways [70]. Both HHV-6 and HCMV infection have been reported to impact profoundly on miRNA expression in infected cells, at the blood and tissue levels [71,72,73,74].

Thus, based on the crucial role of miRNAs expression in the cell alterations linked to fibrosis and the observed effects of HCMV and HHV-6 infection on the expression of SSc-associated fibrosis factors, the present study was addressed to investigate the impact of HCMV and HHV-6A infection on the modulation of miRNA expression in primary human dermal fibroblast cells, which are one of the main relevant targets in SSc pathogenesis and directly involved in fibrosis development.

## 2. Materials and Methods

### 2.1. Primary Human Dermal Fibroblasts

Commercially available primary human dermal fibroblasts (derived from adult skin of single donor) (NHDF-Ad, CC-2511; Lonza, Basel, Switzerland) were cultured as previously described [13] in complete fibroblast cell medium (Fibroblast Cell Basal Medium), supplemented with 2% fetal bovine serum, 0.1% r-human fibroblast growth factor-B, 0.1% insulin, 0.1% gentamicin sulphate/amphotericin-B (Clonetics™ FGM™-2 Bullet Kit™; Lonza, Basel, Switzerland). Sub-cultivation was performed at approximately 80% confluence using the “ReagentPack Subculture Reagent Kit” according to the manufacturer instructions (Lonza, Basel, Switzerland).

### 2.2. Virus Strains and Titration

The same virus stocks were used for all the infections. The reference strain of HCMV (TB40E), kindly provided by Prof. Thomas Mertens (Institute of Virology, Ulm University, Germany), was grown in MRC5 cells, and the viral titre (viral titre: 10^9^ PFU/mL) determined as previously described [75]. In short, ten-fold serial dilutions of HCMV suspensions were used for infection of MRC5 fibroblast monolayers grown in 60 mm-Petri dishes; the titration was performed in triplicate for each dilution. After adsorption (37°C for 2 h), virus *inocula* were removed and replaced with Earle’s modified Minimum Essential Medium with 1% l-glutamine, 1% non-essential aminoacids, 10% fetal calf serum, complemented with 0.6% agarose (Merck KGaA, Darmstadt, Germany) and incubated at 37°C for 7 days. Cell monolayers were stained with the vital dye neutral red for 2 h, then the medium was discarded and cells were fixed with 10% formaline for 10 min at room temperature. Plaques were counted and titres expressed as mean PFU values/mL. The U1102 strain of HHV-6A was propagated in J-Jhan T cells as previously described and contained 10^10^ genome equivalents/mL, determined by real time quantitative PCR (qPCR) as previously described [76].

### 2.3. Virus Infection of Primary Dermal Fibroblasts

HCMV TB40E infections were performed on 90% confluent primary human dermal fibroblasts using a multiplicity of infection (MOI) of 0.1. A MOI of 1.0 was used in the infections with U1102 HHV-6A. Following adsorption (performed by incubation at 37 °C for 2 h), virus *inocula* were removed and replaced with complete fibroblast cell medium. Cells were then incubated at 37°C and cell samples were collected at 0, 4, 7, 10 and 14 days post infection (d.p.i.). Cell samples were collected by scraping and centrifugation at 1000× *g* for 5 min at 4 °C. Cell pellets were then washed in PBS, to eliminate eventual secreted miRNAs, then immediately frozen in liquid nitrogen and kept at −80 °C until use. Two aliquot per sample were prepared, for DNA (10^6^ cells) and RNA (2 × 10^6^ cells) analysis, respectively. Duplicate samples were tested for each series.

### 2.4. DNA Extraction and Quantitative Real-Time PCR (qPCR) Assay

Extraction of total DNA from 10^6^ infected cells was performed by the NucliSENS^®^ EasyMAG^®^ platform (bioMérieux). The extracted DNA was analyzed by the qPCR CMV ELITe MGB^®^ Kit (ELITechGroup, Turin, Italy), able to detect and quantify the human HCMV DNA exon 4 region of the immediate-early (IE)1 gene. The assay was performed following the manufacturer’s instructions in a 7500 Real-time PCR system (ABI PRISM, Applied BioSystems). The results were expressed as DNA copies/mL (logarithmic scale). HHV-6A was quantified by qPCR assay designed to amplify the U94 viral gene, as previously described [76]. Both qPCR assays were performed in a 7500 Real-time PCR system (ABI PRISM, Applied BioSystems) and the results were expressed as DNA genome copy number per mL.

### 2.5. RNA Extraction

The mirVana™ miRNA Isolation Kit (Thermo Fisher Scientific, Waltham, MA, USA) was used to extract small RNAs from 2 × 10^6^ infected and control cells, following the manufacturer’s instructions. The method efficiently isolates small RNA-containing total RNA and enrich the small RNA fraction (<200 nt), allowing increase of sensitivity in downstream analyses. Extracted RNA was checked and quantified by spectrophotometric reading at 260 and 280 nm wavelength, using a Nanodrop reader. Elimination of contaminant DNA was assured by DNase I digestions (Thermo Fisher Scientific, Waltham, MA, USA) and absence of contaminating DNA was assessed by amplifying an aliquot of extracted RNA for human β-actin gene. After verifying that the samples were devoid of contaminating DNA, 10 ng aliquots of total RNA were retrotranscribed by TaqMan Advanced miRNA cDNA Synthesis Kit (Thermo Fisher Scientific, Waltham, MA, USA), according to manufacturer’s instructions. Briefly, mature miRNAs present in the sample were extended on each end by a 3’ poly-A tailing and a 5’ ligation reactions. Then, a Reverse Transcription reaction was performed using universal RT primers, able to recognize the universal sequences added. In addition, in order to improve detection of low-expressing miRNA targets, the cDNA was amplified using the Universal miR-Amp Primers and miR-Amp Master Mix to uniformly increase the amount of cDNA for each target, maintaining the relative differential expression levels. Following retrotranscription, 500 ng of cDNA were used for subsequent analysis.

### 2.6. miRNA Array Analyses

The expression of microRNA in infected or control uninfected cells was analyzed by the TaqMan Advanced miRNA Human A and B Cards 0 (Thermo Fisher Scientific, Waltham, MA, USA), which is a two-card set containing a total of 384 TaqMan^®^ MicroRNA Assays per card allowing identification and accurate quantification of 754 human microRNAs. Total RNA was reverse transcribed following manufacturer’s procedure; cDNAs were loaded on the TaqMan arrays and run on a 7900HT real-time PCR system in accordance with the manufacturer’s procedure. Each card includes five endogenous controls to allow data normalization and two non-human miRNA assays as a negative control. miRNA data were analyzed with SDS RQ Software version 2.4 and with ThermoFisher Cloud app (Thermo Fisher Scientific, Waltham, MA, USA); miRNAs with Ct values ≥ 35 were considered as not expressed and excluded from further analysis. Results are calculated as ΔΔCt values in infected vs. uninfected control cells at each time post infection, and expressed as fold-change values compared to control values after normalizing for the housekeeping endogenous controls. Analysis threshold was put at 2-fold change of up- or- down-modulation. Duplicate samples were analyzed.

### 2.7. Statistical Analyses

A paired t-test was used to determine the significance of differential miRNA expression between control and infected cells. A *p* value < 0.05 was regarded as statistically significant. The miRNet online tool (https://www.mirnet.ca/faces/home.xhtml, accessed on 28 February 2021) was used to analyze the pathways in which deregulated miRNAs were implicated [77]. The database is a comprehensive atlas of miRNA-target interactions that can integrate the information resulting from 11 existing miRNA-target prediction programs (TarBase, miRTarBase, miRecords, miRanda, miR2Disease, HMDD, PhenomiR, SM2miR, PharmacomiR, EpimiR, and starBase). The software uses standard enrichment analysis based on the hypergeometric tests after adjustment for false discovery rate. Heatmaps were generated using the Multiple Experiment Viewer (MEV) tool (http://mev.tm4.org/#/welcome; accessed on 28 February 2021). Analysis of the principal component (PCA) was developed by R CRAN package rgl (https://cran.r-project.org/web/packages/rgl/vignettes/rgl.html, accessed on 28 February 2021) and visualized through Cubemaker online tool (https://tools.altiusinstitute.org/cubemaker/?id=7282c99489401e98#, accessed on 28 February 2021).

## 3. Results

### 3.1. HCMV and HHV-6 Infection in Primary Human Dermal Fibroblasts

The cells were seeded at optimal density 24 h before infection, then infected with HCMV (TB40E strain) or HHV-6 (U1102 strain) at a MOI of 0.1 and 1, respectively. Cell samples were collected at 0, 4, 7, 10 and 14 days post infection (d.p.i.), and total DNA was extracted and processed by specific real time quantitative PCR (qPCR) targeting the HCMV immediate-early (IE)1 gene and the HHV-6 IE U94 gene, respectively. Collected data showed that HCMV DNA amount (copies/mL) increased from day 4 to day 14 p.i. (Table 1). The expected cytopathic effect (CPE) induced by HCMV productive infection in fibroblasts was observable from day 4 p.i. onwards (Figure S1). Results of HHV-6 infection showed, as expected, an initial increase of HHV-6 DNA (from day 4 to 7 p.i.) followed by a gradual decrease till the end of the experiment (14 d.p.i.), suggesting the initial establishment of an active replication rapidly followed by a latent infection (Table 1). No cytopathic effect was observed as a result of HHV-6 fibroblast infection.

### 3.2. Modulation of miRNA Expression in HCMV-Infected Primary Human Dermal Fibroblasts

**Figure 1:**
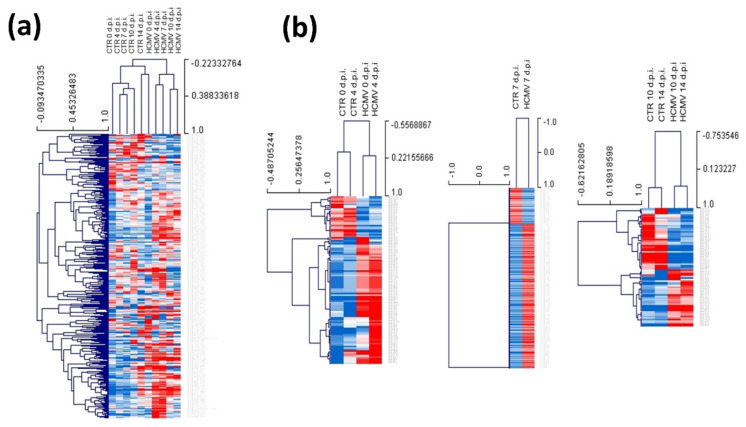
Heatmaps representing the expression of the analyzed microRNA (miRNAs) in human cytomegalovirus (HCMV) infected vs. control uninfected cells (CTR). All up-regulated (blue) and down-regulated (red) values are reported. The colors of the heatmaps were scaled by rows. (**a**) Total deregulated miRNAs at all times post infection (days post-infection; d.p.i.). (**b**) Most altered miRNAs (>±2 fold) subdivided for early (0 and 4 d.p.i.), intermediate (7 d.p.i.), and late (10 and 14 d.p.i.) times post infection.

Infected or control uninfected primary human fibroblasts were collected at 0, 4, 7, 10 and 14 d.p.i. and analyzed for miRNA expression by a TaqMan array allowing identification and quantification of 754 human miRNAs. Figure 1 shows heatmap visualization of the data obtained in HCMV-infected cells, evidencing the great number of miRNAs that were up- or down-modulated upon infection. In detail, just after virus adsorption, a significant alteration of the microRNome (miRNome) expression in the infected fibroblast cells compared to controls was observed. Namely, 85 miRNAs were up-modulated and 128 miRNAs down-modulated, at 0 d.p.i. (*p* < 0.01). At later times p.i., HCMV infection induced the overexpression of 63, 52, 106, and 90 miRNAs and the down-regulation of 170, 207, 116, and 82 miRNAs, at 4, 7, 10, and 14 d.p.i., respectively (*p* < 0.01). 

**Figure 2:**
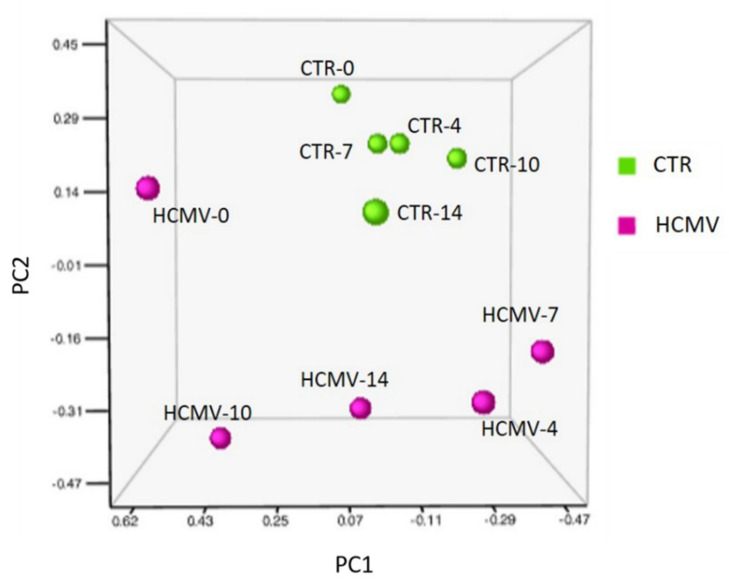
Principal Component Analysis (PCA) of miRNA expression in human cytomegalovirus (HCMV) infected and control uninfected cells (CTR). Tridimensional graph representing miRNA clusters at the different times post infection. HCMV-infected and CTR cells are displayed. Days post-infection (d.p.i.) are indicated by the numbers 0, 4, 7, 10, and 14 after the sample name.

The Principal Component Analysis (PCA) of miRNA expression profiling in HCMV infected and non-infected cells evidenced clearly different clusters of miRNA expression at all the times tested, including the very early ones (0 d.p.i.) (*p* < 0.01) (Figure 2).

Among the significantly up-regulated miRNAs following HCMV infection in vitro, several were highly expressed, with >10 fold difference compared to uninfected controls (Table 2). In particular, just after adsorption, at 0 d.p.i., 17 miRNAs were highly up-regulated (over 10 fold); comparable up-regulation levels were observed for 14 miRNAs at 4 d.p.i., 8 miRNAs at 7 d.p.i., 34 miRNAs at 10 d.p.i., and 28 miRNAs at 14 d.p.i. Some of the HCMV-upregulated miRNAs were previously reported hyper-expressed in fibrotic tissues of SSc patients compared to controls, namely miR-7 (increased at 10 and 14 d.p.i.), miR-20 (increased at 7 and 14 d.p.i.), miR-26a (increased at 14 d.p.i.), miR-92a (increased at 0, 4, 10, and 14 d.p.i.), miR-133 (increased at 7 d.p.i.), miR-140 (increased at 10 d.p.i.), and miR-146b (increased at 0 and 14 d.p.i.). 

Moreover, although to a lesser extent, HCMV infection increased the expression of several other miRNAs reported augmented in fibrosis and SSc patients, including miR-18 (2 fold), miR-31 (2.4 fold), miR-34 (4.8 fold), and miR-130 (4 fold) at 0 d.p.i.; miR-21 (3 fold), miR-29 (3.9 fold), miR-146 (2.9 fold), miR-196 (6.3 fold) at 4 d.p.i.; miR-21 (2 fold), miR-29 (5.1 fold), miR-30 (3.9 fold) at 7 d.p.i.; miR-19 (2.6 fold), miR-20 7.3 fold), miR-130 (4.5 fold), miR-145 (4.6 fold) at 10 d.p.i.; miR-29 2.3 fold), miR-130 (3.4 fold) at 14 d.p.i. 

HCMV infection simultaneously induced the down-regulation of the expression of several miRNAs, some of which appeared strongly decreased (>10 fold) compared to controls). Table 3 summarizes the most significant (*p* < 0.01) down-modulated miRNAs, including 24 miRNAs at 0 d.p.i., 38 at 4 d.p.i., 43 at 7 d.p.i., 25 at 10 d.p.i., and 22 at 14 d.p.i. Interestingly, some of those miRNAs were reportedly shown down-regulated at the tissue level in SSc patients, namely miR-let-7a (significantly decreased at 0, 4, and 7 d.p.i.), miR-10a (decreased at 4, 10, and 14 d.p.i.), miR-29 (decreased at 10 d.p.i.), miR-30b (down-regulated at 0 and 7 d.p.i.), and miR-196a (decreased at 7 and 10 d.p.i.). 

Among the miRNAs previously reported in association with fibrosis and SSc, some appeared regulated by HCMV in an opposite way with respect to what reported, namely miR-29 and miR-196a (up-regulated in HCMV-infected fibroblasts at 0, 4, and 14 d.p.i., but down-modulated in the tissues of SSc patients), and miR-34a (down-regulated at 14 d.p.i. in HCMV infected fibroblasts but up-regulated in SSc patients).

The trend of upregulated and downregulated miRNAs reversed at 7 d.p.i, at which time the lowest number of overexpressed miRNAs and the highest number of underexpressed miRNAs were observed. 

### 3.3. Modulation of miRNA Expression in HHV-6A-Infected Primary Human Dermal Fibroblasts

**Figure 3:**
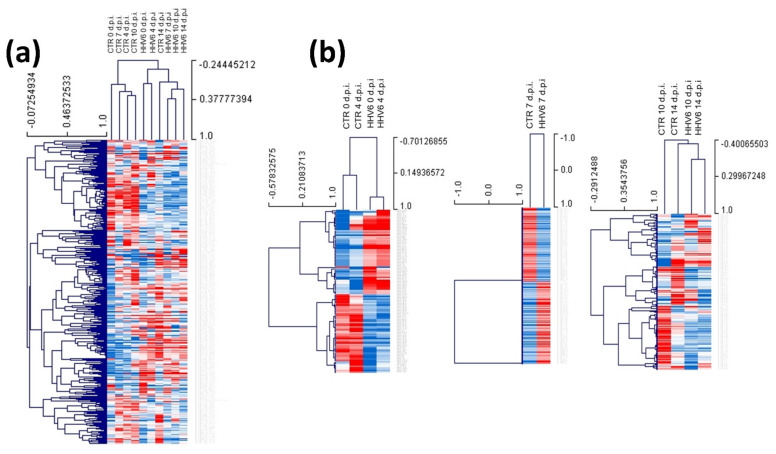
Heatmaps representing the expression of the analyzed miRNA in human herpesvirus 6A (HHV-6A) infected vs. control uninfected cells (CTR). Blue color indicates up-regulation, red color indicates down-regulation. The colors of the heatmaps were scaled by rows. (**a**) Total analyzed miRNAs at all times post infection (days post-infection; d.p.i.). (**b**) Most altered miRNAs (>±2 fold) subdivided into early (0 and 4 d.p.i.), intermediate (7 d.p.i.), and late (10 and 14 d.p.i.) times post infection.

miRNA expression was in parallel analyzed also in HHV-6A infected or control non-infected primary human fibroblasts, collected at the same times post infection as for HCMV (0, 4, 7, 10 and 14 d.p.i.). Figure 3 highlights the strong impact of HHV-6A infection on the cell miRNome alteration in fibroblasts, similar to what previously observed in other cell types [72,73,74]. In detail, 117 miRNAs were up-modulated and 111 miRNAs were down-modulated just after adsorption (0 d.p.i.) (*p* < 0.01). At 4 d.p.i., 106 miRNAs were up-regulated and 101 down-modulated; at 7, 10 and 14 d.p.i. HHV-6A infection induced the overexpression of 100, 150, and 112 miRNAs and the down-regulation of 112, 59, and 35 miRNAs, respectively (*p* < 0.01). 

**Figure 4:**
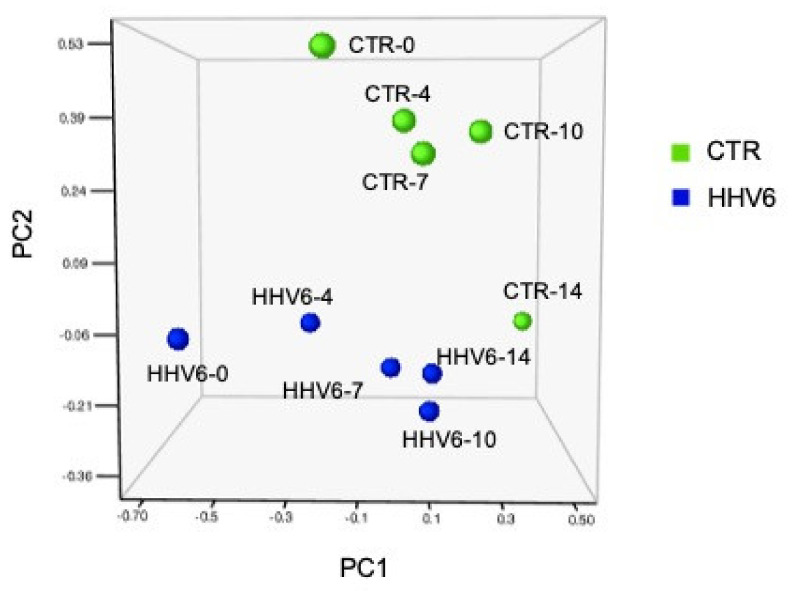
Principal Component Analysis (PCA) of miRNA expression in human herpesvirus 6A (HHV-6A) infected and control uninfected cells (CTR). Tridimensional graph representing miRNA clusters at the different times post infection. HHV-6A-infected and CTR cells are displayed. Days post-infection (d.p.i.) are also indicated after the sample name.

The principal component analysis (PCA) of miRNA expression in HHV-6A infected cells compared to controls, showed very different clusters at all tested times p.i. (Figure 4), confirming the impact of virus infection on the fibroblast cell miRNome.

As shown in Table 4, among the up-regulated miRNAs, some were particularly overexpressed (>10 fold compared to uninfected controls) (*p* < 0.01). In particular, 22 highly up-regulated miRNAs at 0 d.p.i. and 4 d.p.i., 14 miRNAs at 7 d.p.i., 29 miRNAs at 10 d.p.i., and 20 miRNAs at 14 d.p.i. were observed. Some of the up-regulated miRNAs were also found to be overexpressed in tissues from SSc patients, namely miR-7 (increased at 14 d.p.i.), and miR-92a (increased at 0 and 14 d.p.i.).

In addition to those indicated in Table 4, other miRNAs associated with fibrosis were found significantly upregulated (*p* < 0.01) following HHV-6A infection in human primary fibroblasts, although to a lesser extent. These included miR-33b (5.7 fold), miR-31 (2.4 fold), miR-34 (4.8 fold), and miR-130 (4 fold) at 0 d.p.i.; miR-21 (3 fold), miR-29 (3.9 fold), miR-146 (2.9 fold), miR-196 (6.3 fold) at 4 d.p.i.; miR-21 (2 fold), miR-29 (5.1 fold), miR-30 (3.9 fold) at 7 d.p.i.; miR-19 (2.6 fold), miR-20 7.3 fold), miR-130 (4.5 fold), miR-145 (4.6 fold) at 10 d.p.i.; miR-29 2.3 fold), miR-130 (3.4 fold) at 14 d.p.i.. 

Similar to what observed for HCMV infection, also HHV-6A infection induced the down-modulation of several miRNAs in human fibroblasts. Most underexpressed miRNAs (at least 10-fold less than controls) (*p* < 0.01) are indicated in Table 5. They included 20 miRNAs at 0 d.p.i., 13 at 4 d.p.i., 23 at 7 d.p.i., 15 at 10 d.p.i., and 12 at 14 d.p.i. Of those miRNAs, some were reported down-regulated in SSs tissues, such as miR-let-7a (decreased at 4 and 7 d.p.i.), miR-10a (decreased at 10 d.p.i.), miR-20 (decreased at 4 and 10 d.p.i.), miR-34a (decreased at 14 d.p.i.), and miR-193b (decreased at 0, 7, and 10 d.p.i.). 

Conversely, some miRNAs were regulated by HHV-6A infection in an opposite way: miR-193b (increased at 14 d.p.i. in HHV-6A infected cells, and instead under-expressed in SSc tissues), and miR-146b (decreased at 10 d.p.i. in HHV-6A infected fibroblasts but up-regulated in SSc).

**Figure 5:**
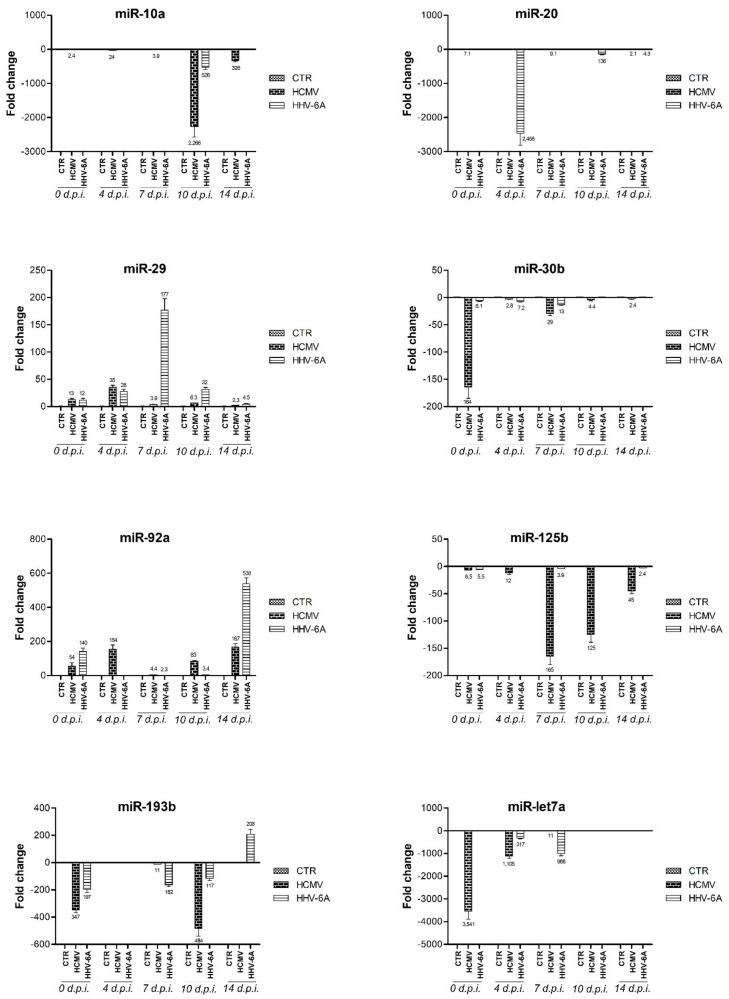
Alteration of fibrosis-associated miRNAs by HCMV and HHV-6A infection in human primary dermal fibroblasts. Results are expressed as mean fold-change ± SD. Mean values are also indicated in the column labels.

MicroRNAs that were deregulated >±10-fold by both HCMV and HHV-6A infection in human primary dermal fibroblasts and involved in tissue fibrosis are depicted in Figure 5. The alterations detected for all the reported miRNAs mimicked what observed in SSc patient tissues. The expression of these miRNAs was mostly altered at all times p.i., although at different extents depending on the virus type and time of collection. For both viruses, it was possible to distinguish early and late effects. In particular, miR-let7a, miR-10a, miR-20, and miR-30b were mostly affected at early times p.i.; miR-20 appeared especially altered at intermediate times (7 d.p.i.); and miR-92a, miR-125b, and miR-193b were affected both in early and late phases of the virus infection. 

### 3.4. Pathways Associated with miRNA Expression in HHV-6A-Infected Primary Human Dermal Fibroblasts

**Figure 6:**
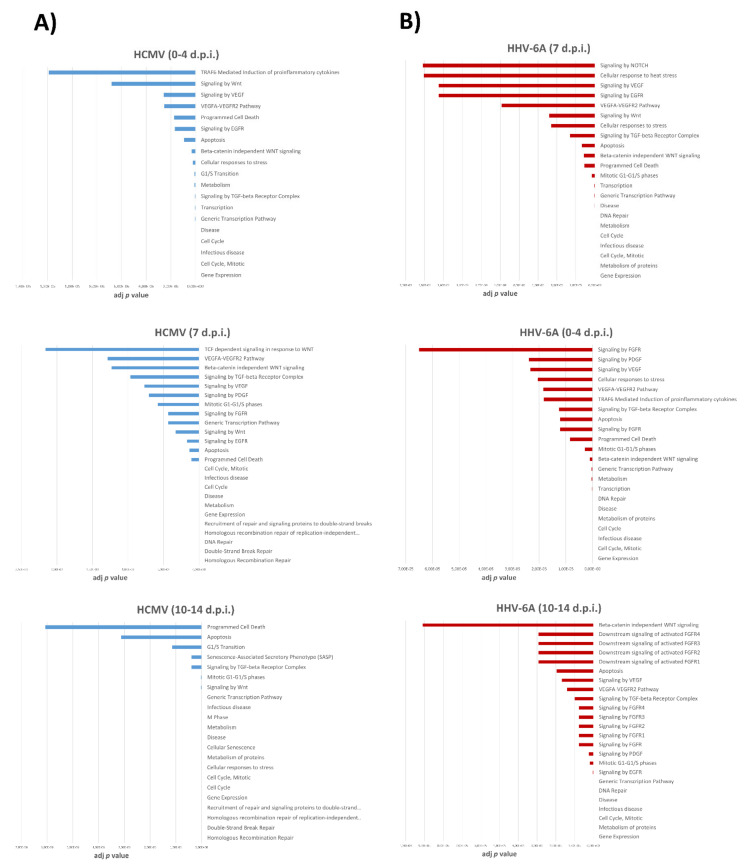
Network connectivity analysis based on differentially expressed miRNAs in HCMV (**A**) and HHV-6A (**B**) infected fibroblasts. Early (0–4 d.p.i.), intermediate (7 d.p.i.) and late (10–14 d.p.i.) effects are shown for both viruses. Results are expressed as adjusted *p* value, indicating how statistically significant is the correlation. A lower *p* value represents a higher association.

The enrichment analysis based on differentially (*p* < 0.01) expressed miRNAs following HCMV and HHV-6A infection in human fibroblasts, revealed several intracellular signals possibly modulated by viruses infection, with potential impact on multiple pathways, as reported in Table S1–S3 (reporting results concerning early, intermediate and late effects of HCMV, respectively) and Tables S4–S6 (regarding HHV-6A early, intermediate and late effects, respectively). Potentially affected signaling pathways are listed based on the relative adjusted *p* value (ranging from 0 to 0.00275), as resulted from network connectivity analysis, representing an indication of how significant is the correlation within a defined module. The pathways most associated with tissue fibrosis, and correlate to virus-induced modulation of cell miRNome, are depicted in Figure 6, reporting early and late effects for both viruses.

As expected, both viruses altered the expression of several miRNAs that are strongly associated with infectious disease, including induction of altered cell cycle, cell transcription and proliferation, and apoptosis. However, miRNA alterations induced by HCMV and HHV-6A infection in human fibroblasts also were significantly correlated (*p* < 0.00001) with specific fibrosis-associated signaling pathways, including those mediated by transformign growth factor (TGF)-β, Wingless/Int (WNT), and β -catenin, vascular endothelial growth factor (VEGF) and its receptor (VEGF-R), epithelial growth factor receptor (EGFR), and fibroblast growth factor receptor (FGFR).

## 4. Discussion

Tissue fibrosis can affect every type of organ or tissue, often leading to organ malfunction [78], but the factors influencing such process are not fully elucidated, including the possible role of infectious agents, besides other possible triggers such as radiation and mechanical injury [1,79]. The lack of validated markers, further renders very difficult the therapeutic approach.

Among infectious agents, the infection by human herpesviruses (particularly HCMV and HHV-6) has been associated with SSc onset/development [19,80,81], but no conclusive data are yet available.

On the other hand the expression of microRNAs (miRNAs) has been found altered in the tissues of SSc patients, suggesting that miRNAs could play a significant role in the disease outbreak and progression.

Consistent with this, the present study aimed to assess the impact of HCMV and HHV-6A infection on the miRNA expression in primary human dermal fibroblasts, to evidence any alteration possibly connected to fibrosis.

The miRNome characterization, obtained by a quantitative real-time PCR array approach to detect and quantify >700 miRNAs, showed that both viruses have the ability to induce a potent modulation of the expression of miRNAs, at very early times p.i. post infection, as a result of the adsorption and entrance of the viruses (0 d.p.i.), as well as at later times (till to 14 d.p.i.). Notably, both HCMV and HHV-6A profoundly affected miRNA expression in infected cells, despite the different kind of infection produced, cytopathic for HCMV and non-cytopathic for HHV-6A, suggesting that miRNome deregulation occurs independently from the induction of other macroscopic alterations in the infected cells.

In particular, HCMV infection induced a profound alteration of cell miRNome, with up to 43 miRNAs highly deregulated (>10 fold; *p* < 0.05) at each time p.i. Similarly, also HHV-6A infection determined a potent up- or down-modulation (>10 fold; *p* < 0.05) of up to 29 miRNAs both at early and late times p.i. Consistently with this significant impact of herpesviruses infection on cellular miRNA expression, PCA analysis revealed distant clusters for each analyzed time, both for HCMV and HHV-6A, suggesting a deep alteration due to virus infection. Unexpectedly, some miRNAs were potently deregulated within 2 h after exposure to the virus, highlighting a very early effect of the virus infection (likely associate with virus adsorption and entering the cells), which is however compatible with the reported kinetic of miRNA activity [82,83,84], and confirms that mature miRNAs are among the fastest produced cell transcripts.

Interestingly, at each time p.i. at least one or more of the altered miRNAs belong to those already recognized as differentially expressed in tissues from SSc patients and potentially associated with fibrosis, fibroblast proliferation and matrix deposition [44]. In particular, both viruses up-regulated miR-7 (up to 917 fold for HCMV and 23 fold for HHV-6A), miR-let-7g (12 fold for HCMV and 23 fold for HHV-6A), miR-92a (up to 167 fold for HCMV and 438 fold for HHV-6A). In this regard, although miR-7 acts against excessive fibrogenesis, it has been suggested that it fails to counteract the pathogenic fibrosis pathways in SSc. In fact, a microarray analysis reported that the expression levels of miR-7 were upregulated in SSc dermal fibroblasts both in vivo and in vitro, probably as a negative feedback against excessive fibrogenesis [85,86]. miR-let-7g was found upregulated in SSc skin tissue [87] and upregulated miR-92a expression was detected in cultured dermal fibroblasts from SSc skin, leading to the down-regulation of Matrix Metalloproteinase (MMP)-1, which results, in turn, in the excessive accumulation of collagen by decreasing collagen degradation [88,89]. Moreover, several data also suggest a close association between miR-92a with pulmonary involvement in SSc [90]. HCMV infection was found to increase miR-146b expression (up to 27 fold), whose levels are known to be higher in SSc skin tissues and fibroblasts, as well as in normal fibroblasts and endothelial cells that were stimulated with SSc patient sera [46].

HCMV and HHV-6A infection were also both associated with down-regulation of miR-let-7a (up to 3451 for HCMV and 988 fold for HHV-6A), miR-10a (up to 2266 fold for HCMV and 526 fold for HHV-6A), and miR-193b (up to 484 fold for HCMV and to 197 fold for HHV-6A). To this regard, literature data report that miR-let-7a downregulation contributes to the abnormally increased expression of type I collagen in SSc [44,64]. miR-10a has been reported to be under-expressed in both SSc skin tissues and primary skin fibroblasts [46]. Finally, miR-193b was down-regulated in SSc fibroblasts and skin sections causing proliferative vasculopathy [50]. HCMV infection also strongly decreased the expression of miR-29 (9 × 10^4^ fold at 10 d.p.i), miR-30b (up to 164 fold), and miR-125b (up to 165 fold). miR-29 represent one of the more extensively studied miRNAs involved in SSc fibrosis because of its potent anti-fibrotic effects, observed in dermal and fibroblasts of SSc patients and in skin, lung, kidney, and cardiac fibrosis [44,59,60]. Also, several data indicate that miR-30b levels were inversely correlated with modified Rodnan skin scores of SSc patients, reflecting disease severity [86,91]. Concerning miR125b, its downregulation has been demonstrated in skin samples and primary dermal fibroblasts obtained from patients with SSc and it was suggested to be a potential anti-fibrotic and anti-apoptotic regulator in SSc [68,87]. HHV-6A infection also induced the strong downregulation of miR-20 (up to 2458 fold at 4 and 10 d.p.i.), which was recently recognized as a key factor in liver fibrosis, since its down-regulation resulted in TGFBR2-activated TGF-β signaling pathway, followed by the activation of macrophage and extracellular matrix (ECM) production [92]. Other miRNAs modulated by HCMV infection and implicated in SSc are miR-34a, miR-140, and miR-196a. However, in these cases, the action of the virus is controversial as it was found to alter their expression in the opposite direction to that observed in the SSc [63,93]. On the other hand, new genes and factors as well as miRNAs are going to be added to the list of those involved in fibrosis, as recently reported for ten new candidate genes for potential therapy during SSc [94], and for the newly identified miR-320a, reported to be crucial in lung fibrosis during SSc [95]. The fibrotic process usually occurs because of the activation of fibroblasts, epithelial-mesenchymal transition and excessive extracellular matrix deposition. Thus, the miRNome map obtained in our study may shed further light on the impact of herpesvirus infection on fibrosis in the next future. 

Notably, by analyzing possible signaling pathways associated with the virus-induced miRNA deregulation, highly statistically significant connections emerged with signaling routes related to fibrosis process. In fact, besides predictable pathways associated with altered cell cycle, transcription, proliferation, and apoptosis, infection by HCMV and HHV-6A also was associated with activation of signaling pathways specifically correlated with induction of fibrosis, including signaling through Wingless/Int (WNT), transforming growth factor (TGF)-β, and β-catenin, whose cross-talk has been recognized as one of the major trigger of fibrotic process. WNT/β-catenin signaling has a key role in tissue development and homeostasis, but is also implicated in abnormal wound repair and fibrogenesis [96]. Aberrant activation of WNT/β-catenin signaling occurs in most fibrotic diseases [97,98] and WNT has been identified as a potential target for therapies against fibrotic disorders, together with β-catenin signaling [97,99]. TGF-β regulates the differentiation of many cell types, but its deregulated activity stimulates extracellular matrix production, and induces tissue fibrosis [100]. Importantly, the signal integration between TGF-β and WNT/β-catenin converges and promotes the fibrosis process [98]. Of note, recently a role of non-coding RNAs, including miRNAs, has been hypothesized in the modulation of TGF-β/WNT pathways [101], suggesting that they may be used for new approaches for diagnosis and treatment of fibrosis. 

## 5. Conclusions

Human fibrotic diseases are a major health problem worldwide and the incomplete knowledge of the fibrosis pathogenesis hampers the identification of suitable markers and targets for therapy. Despite the clinical differences among the fibrotic pathologies, these diseases, including SSc, share the common feature of an uncontrolled and progressive accumulation of fibrotic tissue in affected organs, leading to organ loss of function and death. Although establishing a causal role in complex diseases such as SSc is very difficult, data emerging from the present study suggest that HCMV and/or HHV-6A infection could have a relevant role in the induction of cell fibrosis through miRNA deregulation, and strengthen the notion of miRNAs as promising targets for the development of anti-fibrotic therapies and as biomarkers for SSc diagnosis, assessment of disease progression and differential clinical outcomes.

## Figures and Tables

**Table 1:** Human cytomegalovirus (HCMV) and human herpesvirus 6A (HHV-6A) DNA amounts in infected primary human dermal fibroblasts at the indicated times p.i *.

Times of Infection	HCMV DNA Copies/mL (log_10_)	HHV-6A DNA Copies/mL (log_10_)
0 days	-	-
4 days	5.060 ± 0.004	5.615 ± 0.006
7 days	5.469 ± 0.005	5.990 ± 0.004
10 days	5.778 ± 0.003	5.751 ± 0.001
14 days	7.138 ± 0.004	4.945 ± 0.006

* Results are expressed as mean values of genome copies/mL from two independent experiments ± SD.

**Table 2:** Most up-regulated (>10-fold) miRNAs in human cytomegalovirus (HCMV) infected fibroblasts at 0, 4, 7, 10 and 14 days post-infection (d.p.i.) §¤.

0 d.p.i.	4 d.p.i.	7 d.p.i.	10 d.p.i.	14 d.p.i.
**miR-let-7g (12)**	miR-19b (242)	miR-10b (27)	miR-let 7i (8892)	**miR-7 (917)**
miR-15b (1625)	**miR-29 (35)**	**miR-20 (18)**	**miR-7 (64)**	miR-10b (1897)
miR-27a (20)	miR-32 (23)	miR-95 (11)	miR-17 (68)	miR-18a (2813)
**miR-29 (13)**	**miR-92a (154)**	miR-101 (11)	miR-19a (15)	miR-18b (75)
miR-33a (11)	miR-95 (20)	miR-124 (25)	miR-19b (11)	miR-19b (61)
**miR-92a (54)**	miR-124 (18)	**miR-133 (11)**	miR-33a (11)	**miR-20 (92)**
miR-100 (24,209)	miR-143 (16)	miR-143 (43)	**miR-92a (83)**	**miR-26a (14)**
**miR-146b (27)**	miR-153 (127)	miR-665 (13)	miR-100 (50,264)	miR-26b (20)
miR-153 (32)	miR-181d (11)		miR-124 (15)	**miR-92a (167)**
miR-188 (17)	**miR 196a (18)**		miR-126 (50)	miR-124 (219)
miR-190a (20)	miR-487a (47)		miR-139 (48)	miR-126 (299)
miR-219a (11)	miR-545 (11)		**miR-140 (14)**	miR-132 (14)
miR-381 (113)	miR-573 (24)		miR-143 (58)	**miR-146b (12)**
miR-544a (47)	miR-1275 (13)		miR-153 (61)	miR-192 (21)
miR-545 (18)			miR-181d (72)	**miR-196a (101)**
miR-548a (15)			miR-195 (12)	miR-326 (17)
miR-590 (162)			miR-204 (33)	miR-363 (654)
			miR-210 (11)	miR-431 (32)
			miR-301b (21)	miR-454 (995)
			miR-338 (11)	miR-483 (11)
			miR-363 (130)	miR-486 (10)
			miR-376a (92)	miR-501 (12)
			miR-483 (18)	miR-551b (285)
			miR-524 (15)	miR-589 (17)
			miR-544a (24)	miR-615 (50)
			miR-551b (50)	miR-744 (234)
			miR-570 (11)	miR-766 (28)
			miR-573 (10)	miR-1275 (187)
			miR-589 (12)	
			miR-590 (16)	
			miR-629 (19)	
			miR-744 (31)	
			miR-1226 (17)	
			miR-1275 (43)	

^§^ Fold change is indicated in parentheses. ¤ miRNAs associated to fibrosis in tissues of SSc patients are evidenced in bold.

**Table 3:** Most down-regulated (>10-fold) miRNAs in HCMV-infected human fibroblasts at 0, 4, 7, 10 and 14 days post-infection (d.p.i.) §¤.

0 d.p.i.	4 d.p.i.	7 d.p.i.	10 d.p.i.	14 d.p.i.
**miR-let-7a (3541)**	**miR-let-7a (1105)**	**miR-let-7a (11)**	**miR-10a (2266)**	**miR-10a (326)**
miR-1 (46)	**miR-10a (24)**	miR-let-7f (3704)	miR-20b (585)	miR-24 (25)
miR-10b (252)	miR-15b (490)	miR-let-7i (5 × 10^4^)	miR-24 (193)	**miR-34a (445)**
miR-24 (114)	miR-18a (16)	miR-15b (9686)	miR-29c (9 × 10^4^)	miR-34c (18)
**miR-30b (164)**	miR-18b (432)	miR-23a (24)	miR-34b (10)	miR-99a (469)
miR-124 (64)	miR-20b (137)	miR-24 (34)	miR-99a (662)	miR-103a (89)
miR-126 (296)	miR-23a (1 × 10^5^)	**miR-30b (29)**	miR-100 (54)	miR-122 (32)
miR-139 (12)	miR-24 (72)	miR-33a (180)	miR-122 (935)	**miR-125b (45)**
miR-143 (18)	miR-26a (28)	miR-92b (11)	**miR-125b (125)**	miR-135b (14)
**miR-193b (347)**	miR-27b (3900)	miR-99a (24)	miR-136 (11)	miR-143 (15)
miR-200a (20)	miR-33a (42)	miR-122 (80)	**miR-193b (484)**	miR-148a (47)
miR-200b (89)	miR-34b (33)	miR-1254 (12)	**miR-196a (63)**	miR-181a (156)
miR-331 (21)	miR-99a (69)	**miR-125b (165)**	miR-337 (1681)	miR-199b (26)
miR-369 (110)	miR-122 (14)	miR-135b (12)	miR-422a (58)	miR-200b (123)
miR-374 (415)	**miR-125b (12)**	miR-148a (364)	miR-449b (317)	miR-218 (85)
miR-422 (29)	miR-126 (309)	miR-181d (167)	miR-454 (15)	miR-302d (42)
miR-431 (286)	miR-148a (87)	miR-191 (12)	miR-501 (21)	miR-337 (287)
miR-454 (861)	miR-149 (357)	**miR-193b (11)**	miR-504 (64)	miR-369 (57)
miR-486 (82)	miR-181a (49)	**miR-196b (21)**	miR-542 (51)	miR-449b (18)
miR-496 (74)	miR-299 (1928)	miR-337 (1924)	miR-584 (171)	miR-487a (342)
miR-548 (2,9 × 10^6^)	miR-337 (1668)	miR-362 (10)	miR-615 (64)	miR-542 (21)
miR-585 (1323)	miR-369 (39)	miR-374 (2665)	miR.654 (128)	miR-654 (105)
miR-656 (12)	miR-376a (209)	miR-376a (136)	miR-935 (24)	
miR-942 (35)	miR-411 (32)	miR-409 (13)	miR-1197 (49)	
	miR-431 (126)	miR-422a (34)	miR-1303 (45)	
	miR-524 (80)	miR-432 (10)		
	miR-539 (47)	miR-433 (23)		
	miR-541 (37)	miR-450a (11)		
	miR-542 (17)	miR-454 (13)		
	miR-550a (42)	miR-486 (84)		
	miR-584 (209)	miR-487a (329)		
	miR-654 (1073)	miR-501 (96)		
	miR-665 (28)	miR-504 (115)		
	miR-758 (14)	miR-524 (257)		
	miR-766 (239)	miR-541 (32)		
	miR-889 (191)	miR-542 (355)		
	miR-1197 (58)	miR-548k (95)		
	miR-1270 (24)	miR-550 (284)		
		miR-584 (1195)		
		miR-590 (339)		
		miR-654 (129)		
		miR-889 (513)		
		miR-1197 (139)		

^§^ Fold change is indicated in parentheses; ¤ miRNAs associated to fibrosis in tissues of SSc patients are evidenced in bold.

**Table 4:** Most up-regulated miRNAs (>10-fold) in human herpesvirus 6A (HHV-6A) infected fibroblasts at 0, 4, 7, 10 and 14 days post-infection (d.p.i.) §.

0 d.p.i.	4 d.p.i.	7 d.p.i.	10 d.p.i.	14 d.p.i.
**miR-let-7g (23)**	miR-16 (11)	miR-10b (78)	miR-let-7i (7466)	**miR-7 (23)**
miR-15b (878)	miR-19b (191)	miR-17 (77)	miR-7 (31)	miR-15b (2074)
miR-17 (93)	miR-24 (12)	miR-29a (11)	miR-16 (19)	miR-18a (429)
miR-19a (24)	**miR-29 (28)**	**miR-29 (177)**	miR-17 (95)	miR-19b (4333)
miR-19b (157)	miR-33a (27)	miR-33b (18)	miR-24 (16)	miR-24 (12)
**miR-29 (12)**	miR-33b (27)	miR-143 (208)	**miR-29 (32)**	miR-26b (67)
miR-33a (37)	miR-95 (15)	miR-573 (11)	miR-30e (11)	**miR-92a (538)**
**miR-92a (140)**	miR-101 (12)	miR-615 (14)	miR-33a (26)	miR-126 (53)
miR-143 (61)	miR-132 (113)	miR-665 (39)	miR-33b (16)	miR-192 (11)
miR-153 (16)	miR-153 (308)	miR-935 (10)	miR-139 (26)	**miR-193b (208)**
miR-196b (27)	miR-181d (22)	miR-1248 (21)	miR-143 (112)	miR-376a (587)
miR-363 (10)	miR-363 (17)	miR-1291 (17)	miR-153 (26)	miR-431 (154)
miR-374a (10)	miR-381 (11)	miR-1292 (11)	miR-181d (39)	miR-524 (25)
miR-381 (160)	miR-504 (14)	miR-1303 (23)	miR-210 (10)	miR-585 (758)
miR-524 (77)	miR-542 (14)		miR-376a (63)	miR-589 (47)
miR-544a (22)	miR-573 (14)		miR-544a (37)	miR-615 (174)
miR-550a (19)	miR-590 (16)		miR-548k (15)	miR-629 (41)
miR-573 (23)	miR-625 (11)		miR-550a (24)	miR-744 (28)
miR-590 (10)	miR-629 (10)		miR-573 (60)	miR-766 (37)
miR-605 (11)	miR-665 (14)		miR-585 (32)	miR-935 (11)
miR-1248 (17)	miR-744 (42)		miR-589 (18)	
miR-1290 (12)	miR-708 (11)		miR-590 (11)	
			miR-625 (12)	
			miR-628 (22)	
			miR-675 (47)	
			miR-744 (217)	
			miR-1226 (27)	
			miR-1248 (26)	
			miR-1260a (15)	

^§^ Fold change is indicated in parentheses; ¤ miRNAs associated to fibrosis in tissues of SSc patients are evidenced in bold.

**Table 5:** Most down-regulated (> 10-fold) miRNAs in human herpesvirus 6A (HHV-6A) infected fibroblasts at 0, 4, 7, 10 and 14 days post-infection (d.p.i.) §¤.

0 d.p.i.	4 d.p.i.	7 d.p.i.	10 d.p.i.	14 d.p.i.
miR-1 (39)	**miR-let-7a (317)**	**miR-let-7a (988)**	miR-1 (12)	miR-1 (10)
miR-24 (65)	miR-1 (245)	miR-15b (2585)	**miR-10a (526)**	**miR-34a (336)**
miR-122 (68)	miR-17 (1245)	**miR-30b (13)**	miR-15b (2076)	miR-103a (92)
miR-124 (55)	**miR-18 (2 × 10^4^)**	miR-33a (58)	**miR-20 (136)**	miR-122 (16)
miR-126 (169)	miR-19 (2289)	miR-92b (551)	miR-26b (33)	miR-148a (30)
miR-192 (19)	**miR-20 (2458)**	miR-122 (21)	miR-122 (217)	miR-190a (42)
**miR-193b (197)**	miR-103a (71)	miR-148a (116)	miR-132 (66)	miR-195 (23)
miR-200a (11)	miR-122 (1.2 × 10^4^)	miR-181a (103)	**miR-146b (216)**	miR-337 (184)
miR-200b (75)	miR-124 (1698)	**miR-193b (162)**	**miR-193b (117)**	miR-487a (171)
miR-323 (16)	miR-126 (2251)	miR-196b (92)	miR-330 (67)	miR-500a (12)
miR-340 (341)	miR-139 (1567)	miR-200b (17)	miR-337 (406)	miR-590 (39)
miR-422a (25)	miR-148a (298)	miR-323b (12)	miR-454 (294)	miR-1197 (24)
miR-431 (162)	miR-148a (2213)	miR-337 (619)	miR-605 (24)	
miR-486 (68)		miR-378a (36)	miR-889 (50)	
miR-487a (386)		miR-454 (200)	miR-1303 (11)	
miR-496 (62)		miR-486 (22)		
miR-499a (19)		miR-487a (88)		
miR-548d (2 × 10^6^)		miR-524 (290)		
miR-585 (757)		miR-539 (62)		
miR-889 (292)		miR-541 (10)		
		miR-590 (90)		
		miR-889 (137)		
		miR-1197 (18)		

^§^ Fold change is indicated in parentheses; ¤ miRNAs associated to fibrosis in tissues of SSc patients are evidenced in bold.

## Data Availability

The data presented in this study are openly available in Biostudies international repository website, reference number S-BSST598.

## Supplementary Materials

The following are available online at https://www.mdpi.com/article/10.3390/cells10051060/s1; Tables S1–S3: Pathways potentially altered by miRNA deregulation due to HCMV infection at early (S1), intermediate (S2) and late (S3) times post infection. Tables S4–S6: Pathways potentially altered by miRNA deregulation due to HHV-6A infection at early (S4), intermediate (S5) and late (S6) times post infection. Figure S1: HCMV-induced CPE in infected fibroblasts.

## References

[1] Rosenbloom J., Macarak E., Piera-Velazquez S., Jimenez S.A. (2017). Human Fibrotic Diseases: Current Challenges in Fibrosis Research. Methods Mol. Biol..

[2] Denton C.P., Khanna D. (2017). Systemic sclerosis. Lancet.

[3] Giuggioli D., Colaci M., Cocchiara E., Spinella A., Lumetti F., Ferri C. (2018). From Localized Scleroderma to Systemic Sclerosis: Coexistence or Possible Evolution. Dermatol. Res. Pract..

[4] Sobolewski P., Maslinska M., Wieczorek M., Lagun Z., Malewska A., Roszkiewicz M., Nitskovich R., Szymanska E., Walecka I. (2019). Systemic sclerosis—Multidisciplinary disease: Clinical features and treatment. Reumatologia.

[5] Giacomelli R., Liakouli V., Berardicurti O., Ruscitti P., Di Benedetto P., Carubbi F., Guggino G., Di Bartolomeo S., Ciccia F., Triolo G. (2017). Interstitial lung disease in systemic sclerosis: Current and future treatment. Rheumatol. Int..

[6] Richard N., Hudson M., Wang M., Gyger G., Proudman S., Stevens W., Nikpour M., Baron M., Canadian Scleroderma Research Group, Australian Scleroderma Interest Group (2019). Severe gastrointestinal disease in very early systemic sclerosis is associated with early mortality. Rheumatology.

[7] Gyger G., Baron M. (2015). Systemic Sclerosis: Gastrointestinal Disease and Its Management. Rheum. Dis. Clin. N. Am..

[8] Di Cesare E., Battisti S., Di Sibio A., Cipriani P., Giacomelli R., Liakouli V., Ruscitti P., Masciocchi C. (2013). Early assessment of sub-clinical cardiac involvement in systemic sclerosis (SSc) using delayed enhancement cardiac magnetic resonance (CE-MRI). Eur. J. Radiol..

[9] Woodworth T.G., Suliman Y.A., Li W., Furst D.E., Clements P. (2018). Scleroderma renal crisis and renal involvement in systemic sclerosis. Nat. Rev. Nephrol..

[10] LeRoy E.C., Medsger T.A. (2001). Criteria for the classification of early systemic sclerosis. J. Rheumatol..

[11] Murdaca G., Contatore M., Gulli R., Mandich P., Puppo F. (2016). Genetic factors and systemic sclerosis. Autoimmun. Rev..

[12] Abdulle A.E., Diercks G.F.H., Feelisch M., Mulder D.J., van Goor H. (2018). The Role of Oxidative Stress in the Development of Systemic Sclerosis Related Vasculopathy. Front. Physiol..

[13] Doridot L., Jeljeli M., Chene C., Batteux F. (2019). Implication of oxidative stress in the pathogenesis of systemic sclerosis via inflammation, autoimmunity and fibrosis. Redox Biol..

[14] Arcangeletti M.C., D’Accolti M., Maccari C., Soffritti I., Conto F., Chezzi C., Calderaro A., Ferri C., Caselli E. (2020). Impact of Human Cytomegalovirus and Human Herpesvirus 6 Infection on the Expression of Factors Associated with Cell Fibrosis and Apoptosis: Clues for Implication in Systemic Sclerosis Development. Int. J. Mol. Sci..

[15] Arcangeletti M.C., Maccari C., Vescovini R., Volpi R., Giuggioli D., Sighinolfi G., De Conto F., Chezzi C., Calderaro A., Ferri C. (2018). A Paradigmatic Interplay between Human Cytomegalovirus and Host Immune System: Possible Involvement of Viral Antigen-Driven CD8+ T Cell Responses in Systemic Sclerosis. Viruses.

[16] Caselli E., Soffritti I., D’Accolti M., Bortolotti D., Rizzo R., Sighinolfi G., Giuggioli D., Ferri C. (2019). HHV-6A Infection and Systemic Sclerosis: Clues of a Possible Association. Microorganisms.

[17] Broccolo F., Drago F., Cassina G., Fava A., Fusetti L., Matteoli B., Ceccherini-Nelli L., Sabbadini M.G., Lusso P., Parodi A. (2013). Selective reactivation of human herpesvirus 6 in patients with autoimmune connective tissue diseases. J. Med. Virol..

[18] Broccolo F., Drago F., Paolino S., Cassina G., Gatto F., Fusetti L., Matteoli B., Zaccaria E., Parodi A., Lusso P. (2009). Reactivation of human herpesvirus 6 (HHV-6) infection in patients with connective tissue diseases. J. Clin. Virol..

[19] Broccolo F., Fusetti L., Ceccherini-Nelli L. (2013). Possible role of human herpesvirus 6 as a trigger of autoimmune disease. Sci. World J..

[20] Sehrawat S., Kumar D., Rouse B.T. (2018). Herpesviruses: Harmonious Pathogens but Relevant Cofactors in Other Diseases?. Front. Cell Infect. Microbiol..

[21] Caselli E., Di Luca D. (2007). Molecular biology and clinical associations of Roseoloviruses human herpesvirus 6 and human herpesvirus 7. New Microbiol..

[22] Halenius A., Hengel H. (2014). Human cytomegalovirus and autoimmune disease. Biomed. Res. Int..

[23] Barsotti S., Orlandi M., Codullo V., Di Battista M., Lepri G., Della Rossa A., Guiducci S. (2019). One year in review 2019: Systemic sclerosis. Clin. Exp. Rheumatol..

[24] Sinzger C., Grefte A., Plachter B., Gouw A.S., The T.H., Jahn G. (1995). Fibroblasts, epithelial cells, endothelial cells and smooth muscle cells are major targets of human cytomegalovirus infection in lung and gastrointestinal tissues. J. Gen. Virol..

[25] Mostmans Y., Cutolo M., Giddelo C., Decuman S., Melsens K., Declercq H., Vandecasteele E., De Keyser F., Distler O., Gutermuth J. (2017). The role of endothelial cells in the vasculopathy of systemic sclerosis: A systematic review. Autoimmun. Rev..

[26] Ferri C., Cazzato M., Giuggioli D., Sebastiani M., Magro C. (2002). Systemic sclerosis following human cytomegalovirus infection. Ann. Rheum. Dis..

[27] Lunardi C., Dolcino M., Peterlana D., Bason C., Navone R., Tamassia N., Beri R., Corrocher R., Puccetti A. (2006). Antibodies against human cytomegalovirus in the pathogenesis of systemic sclerosis: A gene array approach. PLoS Med..

[28] Arnson Y., Amital H., Guiducci S., Matucci-Cerinic M., Valentini G., Barzilai O., Maya R., Shoenfeld Y. (2009). The role of infections in the immunopathogensis of systemic sclerosis—Evidence from serological studies. Ann. N. Y. Acad. Sci..

[29] Marou E., Liaskos C., Simopoulou T., Efthymiou G., Dardiotis E., Katsiari C., Scheper T., Meyer W., Hadjigeorgiou G., Bogdanos D.P. (2017). Human cytomegalovirus (HCMV) UL44 and UL57 specific antibody responses in anti-HCMV-positive patients with systemic sclerosis. Clin. Rheumatol..

[30] Efthymiou G., Dardiotis E., Liaskos C., Marou E., Scheper T., Meyer W., Daponte A., Daoussis D., Hadjigeorgiou G., Bogdanos D.P. (2019). A comprehensive analysis of antigen-specific antibody responses against human cytomegalovirus in patients with systemic sclerosis. Clin. Immunol..

[31] Lunardi C., Bason C., Navone R., Millo E., Damonte G., Corrocher R., Puccetti A. (2000). Systemic sclerosis immunoglobulin G autoantibodies bind the human cytomegalovirus late protein UL94 and induce apoptosis in human endothelial cells. Nat. Med..

[32] Namboodiri A.M., Rocca K.M., Pandey J.P. (2004). IgG antibodies to human cytomegalovirus late protein UL94 in patients with systemic sclerosis. Autoimmunity.

[33] Ablashi D., Agut H., Alvarez-Lafuente R., Clark D.A., Dewhurst S., DiLuca D., Flamand L., Frenkel N., Gallo R., Gompels U.A. (2014). Classification of HHV-6A and HHV-6B as distinct viruses. Arch. Virol..

[34] Caruso A., Rotola A., Comar M., Favilli F., Galvan M., Tosetti M., Campello C., Caselli E., Alessandri G., Grassi M. (2002). HHV-6 infects human aortic and heart microvascular endothelial cells, increasing their ability to secrete proinflammatory chemokines. J. Med. Virol..

[35] Caruso A., Caselli E., Fiorentini S., Rotola A., Prandini A., Garrafa E., Saba E., Alessandri G., Cassai E., Di Luca D. (2009). U94 of human herpesvirus 6 inhibits in vitro angiogenesis and lymphangiogenesis. Proc. Natl. Acad. Sci. USA.

[36] Ranger-Rogez S., Vidal E., Liozon F., Denis F. (1994). Primary Sjogren’s syndrome and antibodies to human herpesvirus type 6. Clin. Infect. Dis.

[37] Krueger G.R., Sander C., Hoffmann A., Barth A., Koch B., Braun M. (1991). Isolation of human herpesvirus-6 (HHV-6) from patients with collagen vascular diseases. In Vivo.

[38] Alvarez-Lafuente R., Fernandez-Gutierrez B., de Miguel S., Jover J.A., Rollin R., Loza E., Clemente D., Lamas J.R. (2005). Potential relationship between herpes viruses and rheumatoid arthritis: Analysis with quantitative real time polymerase chain reaction. Ann. Rheum. Dis..

[39] Alvarez-Lafuente R., Martinez A., Garcia-Montojo M., Mas A., De Las Heras V., Dominguez-Mozo M.I., Maria Del Carmen C., Lopez-Cavanillas M., Bartolome M., Gomez de la Concha E. (2010). MHC2TA rs4774C and HHV-6A active replication in multiple sclerosis patients. Eur. J. Neurol..

[40] Caselli E., Zatelli M.C., Rizzo R., Benedetti S., Martorelli D., Trasforini G., Cassai E., degli Uberti E.C., Di Luca D., Dolcetti R. (2012). Virologic and immunologic evidence supporting an association between HHV-6 and Hashimoto’s thyroiditis. PLoS Pathog..

[41] Altorok N., Kahaleh B. (2015). Epigenetics and systemic sclerosis. Semin. Immunopathol..

[42] Altorok N., Almeshal N., Wang Y., Kahaleh B. (2015). Epigenetics, the holy grail in the pathogenesis of systemic sclerosis. Rheumatology.

[43] Rupaimoole R., Slack F.J. (2017). MicroRNA therapeutics: Towards a new era for the management of cancer and other diseases. Nat. Rev. Drug Discov..

[44] Henry T.W., Mendoza F.A., Jimenez S.A. (2019). Role of microRNA in the pathogenesis of systemic sclerosis tissue fibrosis and vasculopathy. Autoimmun. Rev..

[45] Altorok N., Wang Y., Kahaleh B. (2014). Endothelial dysfunction in systemic sclerosis. Curr. Opin. Rheumatol..

[46] Zhou B., Zuo X.X., Li Y.S., Gao S.M., Dai X.D., Zhu H.L., Luo H. (2017). Integration of microRNA and mRNA expression profiles in the skin of systemic sclerosis patients. Sci. Rep..

[47] Yan Q., Chen J., Li W., Bao C., Fu Q. (2016). Corrigendum: Targeting miR-155 to Treat Experimental Scleroderma. Sci. Rep..

[48] Yan Q., Chen J., Li W., Bao C., Fu Q. (2016). Targeting miR-155 to Treat Experimental Scleroderma. Sci. Rep..

[49] Artlett C.M., Sassi-Gaha S., Hope J.L., Feghali-Bostwick C.A., Katsikis P.D. (2017). Mir-155 is overexpressed in systemic sclerosis fibroblasts and is required for NLRP3 inflammasome-mediated collagen synthesis during fibrosis. Arthritis Res. Ther.

[50] Iwamoto N., Vettori S., Maurer B., Brock M., Pachera E., Jungel A., Calcagni M., Gay R.E., Whitfield M.L., Distler J.H. (2016). Downregulation of miR-193b in systemic sclerosis regulates the proliferative vasculopathy by urokinase-type plasminogen activator expression. Ann. Rheum. Dis..

[51] Luo H., Zhu H., Zhou B., Xiao X., Zuo X. (2015). MicroRNA-130b regulates scleroderma fibrosis by targeting peroxisome proliferator-activated receptor gamma. Mod. Rheumatol..

[52] Wermuth P.J., Li Z., Mendoza F.A., Jimenez S.A. (2016). Stimulation of Transforming Growth Factor-beta1-Induced Endothelial-To-Mesenchymal Transition and Tissue Fibrosis by Endothelin-1 (ET-1): A Novel Profibrotic Effect of ET-1. PLoS ONE.

[53] Piera-Velazquez S., Mendoza F.A., Jimenez S.A. (2016). Endothelial to Mesenchymal Transition (EndoMT) in the Pathogenesis of Human Fibrotic Diseases. J. Clin. Med..

[54] Jimenez S.A., Piera-Velazquez S. (2016). Endothelial to mesenchymal transition (EndoMT) in the pathogenesis of Systemic Sclerosis-associated pulmonary fibrosis and pulmonary arterial hypertension. Myth or reality?. Matrix Biol..

[55] Wasson C.W., Abignano G., Hermes H., Malaab M., Ross R.L., Jimenez S.A., Chang H.Y., Feghali-Bostwick C.A., Del Galdo F. (2020). Long non-coding RNA HOTAIR drives EZH2-dependent myofibroblast activation in systemic sclerosis through miRNA 34a-dependent activation of NOTCH. Ann. Rheum. Dis..

[56] Wang J., He W., Xu X., Guo L., Zhang Y., Han S., Shen D. (2017). The mechanism of TGF-beta/miR-155/c-Ski regulates endothelial-mesenchymal transition in human coronary artery endothelial cells. Biosci. Rep..

[57] Katsura A., Suzuki H.I., Ueno T., Mihira H., Yamazaki T., Yasuda T., Watabe T., Mano H., Yamada Y., Miyazono K. (2016). MicroRNA-31 is a positive modulator of endothelial-mesenchymal transition and associated secretory phenotype induced by TGF-beta. Genes Cells.

[58] Guo Y., Li P., Bledsoe G., Yang Z.R., Chao L., Chao J. (2015). Kallistatin inhibits TGF-beta-induced endothelial-mesenchymal transition by differential regulation of microRNA-21 and eNOS expression. Exp. Cell Res..

[59] Peng W.J., Tao J.H., Mei B., Chen B., Li B.Z., Yang G.J., Zhang Q., Yao H., Wang B.X., He Q. (2012). MicroRNA-29: A potential therapeutic target for systemic sclerosis. Expert Opin. Ther. Targets.

[60] Maurer B., Stanczyk J., Jungel A., Akhmetshina A., Trenkmann M., Brock M., Kowal-Bielecka O., Gay R.E., Michel B.A., Distler J.H. (2010). MicroRNA-29, a key regulator of collagen expression in systemic sclerosis. Arthritis Rheum..

[61] Jafarinejad-Farsangi S., Gharibdoost F., Farazmand A., Kavosi H., Jamshidi A., Karimizadeh E., Noorbakhsh F., Mahmoudi M. (2019). MicroRNA-21 and microRNA-29a modulate the expression of collagen in dermal fibroblasts of patients with systemic sclerosis. Autoimmunity.

[62] Makino T., Jinnin M., Etoh M., Yamane K., Kajihara I., Makino K., Ichihara A., Igata T., Sakai K., Fukushima S. (2014). Down-regulation of microRNA-196a in the sera and involved skin of localized scleroderma patients. Eur. J. Dermatol..

[63] Honda N., Jinnin M., Kajihara I., Makino T., Makino K., Masuguchi S., Fukushima S., Okamoto Y., Hasegawa M., Fujimoto M. (2012). TGF-beta-mediated downregulation of microRNA-196a contributes to the constitutive upregulated type I collagen expression in scleroderma dermal fibroblasts. J. Immunol..

[64] Makino K., Jinnin M., Hirano A., Yamane K., Eto M., Kusano T., Honda N., Kajihara I., Makino T., Sakai K. (2013). The downregulation of microRNA let-7a contributes to the excessive expression of type I collagen in systemic and localized scleroderma. J. Immunol..

[65] Gravina G., Wasen C., Garcia-Bonete M.J., Turkkila M., Erlandsson M.C., Toyra Silfversward S., Brisslert M., Pullerits R., Andersson K.M., Katona G. (2017). Survivin in autoimmune diseases. Autoimmun. Rev..

[66] Mahmoudi M.B., Farashahi Yazd E., Gharibdoost F., Sheikhha M.H., Karimizadeh E., Jamshidi A., Mahmoudi M. (2019). Overexpression of apoptosis-related protein, survivin, in fibroblasts from patients with systemic sclerosis. Ir. J. Med. Sci..

[67] Vahidi Manesh P., Farazmand A., Gharibdoost F., Vanaki N., Mostafaei S., Kavosi H., Mahmoudi M.B., Mahmoudi M. (2019). Downregulation of miR-542-3p Contributes to Apoptosis Resistance in Dermal Fibroblasts from Systemic Sclerosis Patients via Survivin Overexpression. Iran. J. Allergy Asthma Immunol..

[68] Kozlova A., Pachera E., Maurer B., Jungel A., Distler J.H.W., Kania G., Distler O. (2019). Regulation of Fibroblast Apoptosis and Proliferation by MicroRNA-125b in Systemic Sclerosis. Arthritis Rheumatol..

[69] Ebrahimiyan H., Gharibdoost F., Aslani S., Kavosi H., Farsad F., Jamshidi A., Mahmoudi M. (2020). microRNAs are potentially regulating the survivin gene in PBMCs from systemic sclerosis patients. Mod. Rheumatol..

[70] Piedade D., Azevedo-Pereira J.M. (2016). The Role of microRNAs in the Pathogenesis of Herpesvirus Infection. Viruses.

[71] Fu M., Gao Y., Zhou Q., Zhang Q., Peng Y., Tian K., Wang J., Zheng X. (2014). Human cytomegalovirus latent infection alters the expression of cellular and viral microRNA. Gene.

[72] Caselli E., D’Accolti M., Soffritti I., Zatelli M.C., Rossi R., Degli Uberti E., Di Luca D. (2017). HHV-6A in vitro infection of thyrocytes and T cells alters the expression of miRNA associated to autoimmune thyroiditis. Virol. J..

[73] Bortolotti D., Soffritti I., D’Accolti M., Gentili V., Di Luca D., Rizzo R., Caselli E. (2020). HHV-6A Infection of Endometrial Epithelial Cells Affects miRNA Expression and Trophoblast Cell Attachment. Reprod. Sci..

[74] Rizzo R., Soffritti I., D’Accolti M., Bortolotti D., Di Luca D., Caselli E. (2017). HHV-6A/6B Infection of NK Cells Modulates the Expression of miRNAs and Transcription Factors Potentially Associated to Impaired NK Activity. Front. Microbiol..

[75] Arcangeletti M.C., De Conto F., Ferraglia F., Pinardi F., Gatti R., Orlandini G., Calderaro A., Motta F., Medici M.C., Martinelli M. (2003). Human cytomegalovirus proteins PP65 and IEP72 are targeted to distinct compartments in nuclei and nuclear matrices of infected human embryo fibroblasts. J. Cell. Biochem..

[76] Caselli E., Bracci A., Galvan M., Boni M., Rotola A., Bergamini C., Cermelli C., Dal Monte P., Gompels U.A., Cassai E. (2006). Human herpesvirus 6 (HHV-6) U94/REP protein inhibits betaherpesvirus replication. Virology.

[77] Chang L., Zhou G., Soufan O., Xia J. (2020). miRNet 2.0: Network-based visual analytics for miRNA functional analysis and systems biology. Nucleic Acids Res..

[78] Distler J.H.W., Gyorfi A.H., Ramanujam M., Whitfield M.L., Konigshoff M., Lafyatis R. (2019). Shared and distinct mechanisms of fibrosis. Nat. Rev. Rheumatol..

[79] Gardet A., Zheng T.S., Viney J.L. (2013). Genetic architecture of human fibrotic diseases: Disease risk and disease progression. Front. Pharmacol..

[80] Grossman C., Dovrish Z., Shoenfeld Y., Amital H. (2011). Do infections facilitate the emergence of systemic sclerosis?. Autoimmun. Rev..

[81] Randone S.B., Guiducci S., Cerinic M.M. (2008). Systemic sclerosis and infections. Autoimmun. Rev..

[82] Ando H., Hirose M., Kurosawa G., Impey S., Mikoshiba K. (2017). Time-lapse imaging of microRNA activity reveals the kinetics of microRNA activation in single living cells. Sci. Rep..

[83] Reichholf B., Herzog V.A., Fasching N., Manzenreither R.A., Sowemimo I., Ameres S.L. (2019). Time-Resolved Small RNA Sequencing Unravels the Molecular Principles of MicroRNA Homeostasis. Mol. Cell.

[84] Zlotorynski E. (2019). Insights into the kinetics of microRNA biogenesis and turnover. Nat. Rev. Mol. Cell Biol..

[85] Kajihara I., Jinnin M., Yamane K., Makino T., Honda N., Igata T., Masuguchi S., Fukushima S., Okamoto Y., Hasegawa M. (2012). Increased accumulation of extracellular thrombospondin-2 due to low degradation activity stimulates type I collagen expression in scleroderma fibroblasts. Am. J. Pathol..

[86] Li Y., Huang J., Guo M., Zuo X. (2015). MicroRNAs Regulating Signaling Pathways: Potential Biomarkers in Systemic Sclerosis. Genom. Proteom. Bioinform..

[87] Li H., Yang R., Fan X., Gu T., Zhao Z., Chang D., Wang W. (2012). MicroRNA array analysis of microRNAs related to systemic scleroderma. Rheumatol. Int..

[88] Kuroda K., Shinkai H. (1997). Gene expression of types I and III collagen, decorin, matrix metalloproteinases and tissue inhibitors of metalloproteinases in skin fibroblasts from patients with systemic sclerosis. Arch. Dermatol. Res..

[89] Sing T., Jinnin M., Yamane K., Honda N., Makino K., Kajihara I., Makino T., Sakai K., Masuguchi S., Fukushima S. (2012). microRNA-92a expression in the sera and dermal fibroblasts increases in patients with scleroderma. Rheumatology.

[90] Bagnato G., Roberts W.N., Roman J., Gangemi S. (2017). A systematic review of overlapping microRNA patterns in systemic sclerosis and idiopathic pulmonary fibrosis. Eur. Respir. Rev..

[91] Tanaka S., Suto A., Ikeda K., Sanayama Y., Nakagomi D., Iwamoto T., Suzuki K., Kambe N., Matsue H., Matsumura R. (2013). Alteration of circulating miRNAs in SSc: miR-30b regulates the expression of PDGF receptor beta. Rheumatology.

[92] Fu X., Qie J., Fu Q., Chen J., Jin Y., Ding Z. (2020). miR-20a-5p/TGFBR2 Axis Affects Pro-inflammatory Macrophages and Aggravates Liver Fibrosis. Front. Oncol..

[93] Zhang L., Wu H., Zhao M., Lu Q. (2020). Meta-analysis of differentially expressed microRNAs in systemic sclerosis. Int. J. Rheum. Dis..

[94] Kerick M., Gonzalez-Serna D., Carnero-Montoro E., Teruel M., Acosta-Herrera M., Makowska Z., Buttgereit A., Babaei S., Barturen G., Lopez-Isac E. (2021). eQTL analysis in systemic sclerosis identifies new candidate genes associated with multiple aspects of disease pathology. Arthritis Rheumatol..

[95] Li Y., Huang J., Hu C., Zhou J., Xu D., Hou Y., Wu C., Zhao J., Li M., Zeng X. (2021). MicroRNA-320a: An important regulator in the fibrotic process in interstitial lung disease of systemic sclerosis. Arthritis Res. Ther..

[96] Lam A.P., Gottardi C.J. (2011). beta-catenin signaling: A novel mediator of fibrosis and potential therapeutic target. Curr. Opin. Rheumatol..

[97] Cao H., Wang C., Chen X., Hou J., Xiang Z., Shen Y., Han X. (2018). Inhibition of Wnt/beta-catenin signaling suppresses myofibroblast differentiation of lung resident mesenchymal stem cells and pulmonary fibrosis. Sci. Rep..

[98] Piersma B., Bank R.A., Boersema M. (2015). Signaling in Fibrosis: TGF-beta, WNT, and YAP/TAZ Converge. Front. Med..

[99] Burgy O., Konigshoff M. (2018). The WNT signaling pathways in wound healing and fibrosis. Matrix Biol..

[100] Contreras O., Soliman H., Theret M., Rossi F.M.V., Brandan E. (2020). TGF-beta-driven downregulation of the transcription factor TCF7L2 affects Wnt/beta-catenin signaling in PDGFRalpha(+) fibroblasts. J. Cell Sci..

[101] Yousefi F., Shabaninejad Z., Vakili S., Derakhshan M., Movahedpour A., Dabiri H., Ghasemi Y., Mahjoubin-Tehran M., Nikoozadeh A., Savardashtaki A. (2020). TGF-beta and WNT signaling pathways in cardiac fibrosis: Non-coding RNAs come into focus. Cell Commun. Signal..

