# Supplementary material for: Modulation of microRNome by Human Cytomegalovirus and Human Herpesvirus 6 Infection in Human Dermal Fibroblasts: Possible Significance in the Induction of Fibrosis in Systemic Sclerosis
Source: Cells. 2021 Apr 29;10(5):1060. doi: 10.3390/cells10051060 (PMC8146000; doi:10.3390/cells10051060)
Supplement: Supplementary file 1 [file cells-10-01060-s001.zip › Table S1.pdf]

Table S1. Enrichment analysis based on differentially expressed miRNAs at early times post HCMV infection (0 and 4 d.p.i.).

**Pathways**	***p* val**	**adj *p* val**
Gene Expression	3.04E-37	3.04E-35
Cell Cycle, Mitotic	3.19E-20	1.6E-18
Infectious disease	1.36E-16	4.53E-15
Cell Cycle	1.05E-14	2.63E-13
Disease	3.05E-13	6.1E-12
HIV Infection	7.76E-13	1.12E-11
Metabolism of proteins	7.84E-13	1.12E-11
Mitotic G1-G1/S phases	8.74E-11	1.09E-09
Mitotic Metaphase and Anaphase	1.01E-10	1.12E-09
Mitotic Anaphase	1.26E-10	1.26E-09
M Phase	2.21E-10	2.01E-09
Membrane Trafficking	5.09E-10	3.92E-09
Translation	5.09E-10	3.92E-09
HIV Life Cycle	6.67E-10	4.49E-09
Separation of Sister Chromatids	7.01E-10	4.49E-09
S Phase	7.18E-10	4.49E-09
Late Phase of HIV Life Cycle	8.17E-10	4.81E-09
Signalling by NGF	4E-09	2.22E-08
Generic Transcription Pathway	5.68E-09	2.99E-08
Transcription	6.97E-09	3.49E-08
Signaling by TGF-beta Receptor Complex	1.06E-08	5.05E-08
Influenza Infection	1.45E-08	6.59E-08
Metabolism	1.52E-08	6.61E-08
G1/S Transition	1.82E-08	7.58E-08
Mitotic Prometaphase	3.62E-08	1.38E-07
DNA Replication	3.62E-08	1.38E-07
Host Interactions of HIV factors	3.72E-08	1.38E-07
Influenza Life Cycle	4.28E-08	1.53E-07
Cellular responses to stress	6.43E-08	2.14E-07
Signaling by the B Cell Receptor (BCR)	6.43E-08	2.14E-07
Diseases of signal transduction	8.55E-08	2.76E-07
Developmental Biology	9.39E-08	2.93E-07
Influenza Viral RNA Transcription and Replication	1.01E-07	2.97E-07
Beta-catenin independent WNT signaling	1.01E-07	2.97E-07
Synthesis of DNA	1.13E-07	3.23E-07
Signaling by Rho GTPases	1.27E-07	3.53E-07
Mitotic G2-G2/M phases	1.55E-07	4.19E-07
Downstream signaling events of B Cell Receptor (BCR)	1.6E-07	4.21E-07
Cyclin E associated events during G1/S transition	1.78E-07	4.56E-07
Vesicle-mediated transport	1.87E-07	4.68E-07

Resolution of Sister Chromatid Cohesion	2.22E-07	5.39E-07
SRP-dependent cotranslational protein targeting to membrane	2.37E-07	5.39E-07
G2/M Transition	2.37E-07	5.39E-07
GTP hydrolysis and joining of the 60S ribosomal subunit	2.37E-07	5.39E-07
L13a-mediated translational silencing of Ceruloplasmin expression	2.93E-07	6.1E-07
3' -UTR-mediated translational regulation	2.93E-07	6.1E-07
Cyclin A:Cdk2-associated events at S phase entry	2.98E-07	6.1E-07
Eukaryotic Translation Initiation	2.99E-07	6.1E-07
Cap-dependent Translation Initiation	2.99E-07	6.1E-07
CLEC7A (Dectin-1) signaling	3.33E-07	6.66E-07
Cell Cycle Checkpoints	3.55E-07	6.96E-07
Apoptosis	4.77E-07	9.17E-07
PCP/CE pathway	8.08E-07	1.48E-06
TRIF-mediated TLR3/TLR4 signaling	8.08E-07	1.48E-06
DNA Repair	8.13E-07	1.48E-06
Signaling by EGFR	9.37E-07	1.67E-06
Programmed Cell Death	9.91E-07	1.72E-06
RNA Polymerase I, RNA Polymerase III, and Mitochondrial Transcription	0.000001	1.72E-06
RHO GTPases Activate Formins	1.03E-06	1.75E-06
VEGFA-VEGFR2 Pathway	1.51E-06	2.52E-06
Signaling by VEGF	1.57E-06	2.57E-06
Organelle biogenesis and maintenance	1.71E-06	2.76E-06
SCF(Skp2)-mediated degradation of p27/p21	1.78E-06	2.83E-06
NGF signalling via TRKA from the plasma membrane	2.29E-06	3.58E-06
Formation of a pool of free 40S subunits	2.38E-06	3.66E-06
Axon guidance	2.51E-06	3.8E-06
Cellular response to heat stress	2.56E-06	3.82E-06
Processing of Capped Intron-Containing Pre-mRNA	3.38E-06	4.97E-06
Hedgehog 'on' state	0.000004	5.53E-06
M/G1 Transition	0.000004	5.53E-06
DNA Replication Pre-Initiation	0.000004	5.53E-06
Removal of licensing factors from origins	4.04E-06	5.53E-06
Regulation of DNA replication	4.04E-06	5.53E-06
Autodegradation of Cdh1 by Cdh1:APC/C	4.26E-06	5.76E-06
Regulation of mRNA stability by proteins that bind AU-rich elements	4.38E-06	5.84E-06
Nonsense-Mediated Decay (NMD)	4.64E-06	6.03E-06
Nonsense Mediated Decay (NMD) enhanced by the Exon Junction Complex (EJC)	4.64E-06	6.03E-06
MyD88-independent TLR3/TLR4 cascade	4.98E-06	6.3E-06
Toll Like Receptor 3 (TLR3) Cascade	4.98E-06	6.3E-06
Signaling by Wnt	5.45E-06	6.81E-06
C-type lectin receptors (CLRs)	6.13E-06	7.57E-06
Orc1 removal from chromatin	6.39E-06	7.7E-06
Switching of origins to a post-replicative state	6.39E-06	7.7E-06

Activated TLR4 signalling	6.64E-06	7.81E-06
Nonsense Mediated Decay (NMD) independent of the Exon Junction Complex (EJC)	6.64E-06	7.81E-06
mRNA Splicing - Major Pathway	7.2E-06	8.24E-06
mRNA Splicing	7.2E-06	8.24E-06
Fc epsilon receptor (FCERI) signaling	7.25E-06	8.24E-06
Signaling by Interleukins	8.29E-06	9.31E-06
Regulation of APC/C activators between G1/S and early anaphase	9.59E-06	1.07E-05
Eukaryotic Translation Elongation	9.97E-06	1.1E-05
Activation of NF-kappaB in B cells	1.01E-05	1.1E-05
TRAF6 Mediated Induction of proinflammatory cytokines	1.11E-05	1.19E-05
Post-translational protein modification	1.16E-05	1.21E-05
MyD88:Mal cascade initiated on plasma membrane	1.19E-05	1.21E-05
Toll Like Receptor TLR1:TLR2 Cascade	1.19E-05	1.21E-05
Toll Like Receptor TLR6:TLR2 Cascade	1.19E-05	1.21E-05
Toll Like Receptor 2 (TLR2) Cascade	1.19E-05	1.21E-05
Regulation of PLK1 Activity at G2/M Transition	1.54E-05	1.56E-05
Assembly of the pre-replicative complex	1.58E-05	1.58E-05
