# Supplementary material for: Modulation of microRNome by Human Cytomegalovirus and Human Herpesvirus 6 Infection in Human Dermal Fibroblasts: Possible Significance in the Induction of Fibrosis in Systemic Sclerosis
Source: Cells. 2021 Apr 29;10(5):1060. doi: 10.3390/cells10051060 (PMC8146000; doi:10.3390/cells10051060)
Supplement: Supplementary file 1 [file cells-10-01060-s001.zip › Table S2.pdf]

Table S2. Enrichment analysis based on differentially expressed miRNAs at intermediate time post HCMV infection (7 d.p.i.)

**Pathways**	***p* val**	**adj *p* val**
Homologous Recombination Repair	0	0
Double-Strand Break Repair	0	0
DNA Repair	0	0
Homologous recombination repair of replication-independent double-strand breaks	0	0
Recruitment of repair and signaling proteins to double-strand breaks	0	0
Gene Expression	4.42E-17	7.37E-16
Metabolism	1.36E-11	1.94E-10
Disease	2.88E-10	3.6E-09
Cell Cycle	1.57E-09	1.74E-08
Infectious disease	3.55E-09	3.55E-08
Cell Cycle, Mitotic	6.66E-09	6.05E-08
Metabolism of proteins	1.37E-08	1.14E-07
HIV Infection	7.95E-08	6.12E-07
Organelle biogenesis and maintenance	4.46E-07	3.07E-06
Developmental Biology	4.6E-07	3.07E-06
HIV Life Cycle	1.15E-06	6.94E-06
Platelet activation, signaling and aggregation	1.18E-06	6.94E-06
Adaptive Immune System	2.36E-06	1.31E-05
Immune System	2.89E-06	1.52E-05
Late Phase of HIV Life Cycle	3.19E-06	1.6E-05
Diseases of signal transduction	4.82E-06	2.3E-05
Membrane Trafficking	6.95E-06	3.16E-05
Vesicle-mediated transport	1.29E-05	5.61E-05
Post-translational protein modification	1.36E-05	5.67E-05
Signalling by NGF	1.64E-05	6.56E-05
Programmed Cell Death	2.83E-05	0.000109
Apoptosis	3.69E-05	0.000137
Signaling by the B Cell Receptor (BCR)	4.06E-05	0.000145
NGF signalling via TRKA from the plasma membrane	4.41E-05	0.000152
Signaling by EGFR	5.06E-05	0.000169
Cell Cycle Checkpoints	0.000061	0.000197
Downstream signaling events of B Cell Receptor (BCR)	7.09E-05	0.000222
Assembly of the primary cilium	7.71E-05	0.000228
M Phase	7.76E-05	0.000228
S Phase	0.000107	0.000306
Transcription	0.000117	0.000325
Mitochondrial translation	0.000122	0.000329
Signaling by Wnt	0.000125	0.000329
Cellular responses to stress	0.000178	0.000433
Generic Transcription Pathway	0.000186	0.000433

Mitotic G2-G2/M phases	0.000187	0.000433
Signaling by ERBB2	0.000193	0.000433
Downstream signal transduction	0.00021	0.000433
Signaling by FGFR	0.00021	0.000433
Mitochondrial translation initiation	0.000223	0.000433
Mitochondrial translation elongation	0.000223	0.000433
Mitochondrial translation termination	0.000223	0.000433
G2/M Transition	0.000225	0.000433
Metabolism of lipids and lipoproteins	0.000253	0.000477
Translation	0.000317	0.000582
Hemostasis	0.000321	0.000582
Host Interactions of HIV factors	0.000332	0.000582
Mitotic G1-G1/S phases	0.000332	0.000582
Axon guidance	0.000346	0.000597
SUMOylation	0.000368	0.000624
Mitotic Metaphase and Anaphase	0.000382	0.000637
MyD88-independent TLR3/TLR4 cascade	0.000408	0.000656
Toll Like Receptor 3 (TLR3) Cascade	0.000408	0.000656
Mitotic Anaphase	0.000413	0.000656
TRIF-mediated TLR3/TLR4 signaling	0.000451	0.000705
Signaling by PDGF	0.00046	0.000708
Signaling by VEGF	0.000515	0.000769
DNA Replication	0.000515	0.000769
Downstream signaling of activated FGFR	0.000561	0.00079
SUMOylation of DNA damage response and repair proteins	0.00061	0.000836
SUMO E3 ligases SUMOylate target proteins	0.00061	0.000836
DAP12 signaling	0.00071	0.000957
Asparagine N-linked glycosylation	0.000718	0.000957
Toll Like Receptor 4 (TLR4) Cascade	0.000743	0.000968
Signaling by TGF-beta Receptor Complex	0.000745	0.000968
Separation of Sister Chromatids	0.000766	0.000982
Synthesis of DNA	0.000814	0.00103
Fc epsilon receptor (FCERI) signaling	0.000832	0.00104
Activated TLR4 signalling	0.000977	0.001206
Beta-catenin independent WNT signaling	0.00101	0.001232
VEGFA-VEGFR2 Pathway	0.00107	0.001289
Toll-Like Receptors Cascades	0.00119	0.001417
Signaling by Hedgehog	0.0013	0.001529
Transcription of the HIV genome	0.00136	0.001581
Anchoring of the basal body to the plasma membrane	0.0014	0.001609
RNA Polymerase II Transcription	0.00154	0.00175
RNA Polymerase II Pre-transcription Events	0.00167	0.001867
Regulation of mRNA stability by proteins that bind AU-rich elements	0.00168	0.001867

Response to elevated platelet cytosolic Ca2+	0.00184	0.002022
TCF dependent signaling in response to WNT	0.00199	0.002161
C-type lectin receptors (CLRs)	0.00201	0.002161
Metabolism of amino acids and derivatives	0.00208	0.002213
Metabolism of carbohydrates	0.00217	0.00226
Influenza Infection	0.00217	0.00226
Cyclin E associated events during G1/S transition	0.00225	0.00232
G1/S Transition	0.00233	0.002378
Autodegradation of Cdh1 by Cdh1:APC/C	0.00249	0.002515
Processing of Capped Intron-Containing Pre-mRNA	0.00275	0.00275
