# Supplementary material for: Modulation of microRNome by Human Cytomegalovirus and Human Herpesvirus 6 Infection in Human Dermal Fibroblasts: Possible Significance in the Induction of Fibrosis in Systemic Sclerosis
Source: Cells. 2021 Apr 29;10(5):1060. doi: 10.3390/cells10051060 (PMC8146000; doi:10.3390/cells10051060)
Supplement: Supplementary file 1 [file cells-10-01060-s001.zip › Table S3.pdf]

Table S3. Enrichment analysis based on differentially expressed miRNAs at late times post HCMV infection (10 and 14 d.p.i.)

**Pathways**	***p* val**	**adj *p* val**
Homologous Recombination Repair	0	0
Double-Strand Break Repair	0	0
Homologous recombination repair of replication-independent double-strand breaks	0	0
Recruitment of repair and signaling proteins to double-strand breaks	0	0
Gene Expression	6.44E-41	1.29E-39
Cell Cycle	5.35E-25	8.92E-24
Cell Cycle, Mitotic	4.42E-24	6.31E-23
Cellular responses to stress	1.77E-19	2.21E-18
Metabolism of proteins	1.42E-14	1.58E-13
Cellular Senescence	2.34E-14	2.23E-13
Disease	2.45E-14	2.23E-13
Metabolism	3.24E-13	2.7E-12
M Phase	6.66E-13	5.12E-12
Infectious disease	6.03E-11	4.31E-10
Generic Transcription Pathway	9.03E-11	6.02E-10
RHO GTPase Effectors	5.03E-10	3.14E-09
Epigenetic regulation of gene expression	1.19E-09	7E-09
Signaling by Rho GTPases	2.57E-09	1.43E-08
Post-translational protein modification	3.79E-09	1.99E-08
Oxidative Stress Induced Senescence	4.47E-09	2.24E-08
Signaling by Wnt	4.85E-09	2.31E-08
Mitotic G1-G1/S phases	5.54E-09	2.52E-08
HIV Infection	7.07E-09	3.07E-08
Mitotic G2-G2/M phases	1.12E-08	4.67E-08
G2/M Transition	1.69E-08	6.65E-08
DNA Repair	1.73E-08	6.65E-08
S Phase	3.26E-08	1.21E-07
Translation	4.09E-08	1.46E-07
TCF dependent signaling in response to WNT	4.27E-08	1.47E-07
Negative epigenetic regulation of rRNA expression	5.02E-08	1.67E-07
Mitotic Metaphase and Anaphase	6.19E-08	2E-07
Mitotic Anaphase	7.35E-08	2.3E-07
Cell Cycle Checkpoints	7.71E-08	2.34E-07
NoRC negatively regulates rRNA expression	9.7E-08	2.85E-07
Chromatin modifying enzymes	1.25E-07	3.47E-07
Chromatin organization	1.25E-07	3.47E-07
Signaling by TGF-beta Receptor Complex	1.5E-07	3.95E-07
Senescence-Associated Secretory Phenotype (SASP)	1.5E-07	3.95E-07
Asparagine N-linked glycosylation	2.38E-07	6.1E-07
HIV Life Cycle	2.45E-07	6.13E-07
Separation of Sister Chromatids	2.88E-07	7.02E-07

Organelle biogenesis and maintenance	3.92E-07	9.33E-07
G1/S Transition	4.92E-07	1.14E-06
Influenza Infection	5.65E-07	1.28E-06
SUMOylation	6.6E-07	1.47E-06
DNA Replication	8.73E-07	1.9E-06
Mitotic Prophase	0.000001	2.13E-06
Respiratory electron transport	1.23E-06	2.56E-06
Influenza Life Cycle	1.39E-06	2.84E-06
Late Phase of HIV Life Cycle	1.42E-06	2.84E-06
Apoptosis	1.65E-06	3.13E-06
Eukaryotic Translation Initiation	1.66E-06	3.13E-06
Cap-dependent Translation Initiation	1.66E-06	3.13E-06
SUMOylation of DNA damage response and repair proteins	1.86E-06	3.38E-06
SUMO E3 ligases SUMOylate target proteins	1.86E-06	3.38E-06
RHO GTPases activate PKNs	2.09E-06	3.73E-06
Synthesis of DNA	2.26E-06	3.95E-06
Transcriptional regulation by small RNAs	2.29E-06	3.95E-06
Cellular response to heat stress	2.81E-06	4.7E-06
Influenza Viral RNA Transcription and Replication	2.82E-06	4.7E-06
Membrane Trafficking	3.68E-06	6.03E-06
Programmed Cell Death	3.77E-06	6.08E-06
Axon guidance	4.4E-06	6.98E-06
Regulation of PLK1 Activity at G2/M Transition	4.51E-06	7.05E-06
Diseases of signal transduction	4.99E-06	7.68E-06
GTP hydrolysis and joining of the 60S ribosomal subunit	5.7E-06	8.64E-06
L13a-mediated translational silencing of Ceruloplasmin expression	6.8E-06	0.00001
3' -UTR-mediated translational regulation	6.8E-06	0.00001
Transcription	6.94E-06	1.01E-05
TRIF-mediated TLR3/TLR4 signaling	9.85E-06	1.41E-05
Assembly of the primary cilium	1.05E-05	1.48E-05
Developmental Biology	1.45E-05	2.01E-05
G2/M Checkpoints	0.000015	2.05E-05
MyD88:Mal cascade initiated on plasma membrane	1.76E-05	2.29E-05
Toll Like Receptor TLR1:TLR2 Cascade	1.76E-05	2.29E-05
Toll Like Receptor TLR6:TLR2 Cascade	1.76E-05	2.29E-05
Toll Like Receptor 2 (TLR2) Cascade	1.76E-05	2.29E-05
Activated TLR4 signalling	1.92E-05	2.46E-05
Processing of Capped Intron-Containing Pre-mRNA	1.97E-05	2.49E-05
Nonsense-Mediated Decay (NMD)	2.16E-05	2.67E-05
Nonsense Mediated Decay (NMD) enhanced by the Exon Junction Complex (EJC)	2.16E-05	2.67E-05
Host Interactions of HIV factors	0.000024	2.93E-05
SRP-dependent cotranslational protein targeting to membrane	2.55E-05	3.07E-05
Cyclin E associated events during G1/S transition	2.68E-05	3.15E-05

Regulatory RNA pathways	2.71E-05	3.15E-05
Mitotic Prometaphase	2.71E-05	3.15E-05
Recruitment of mitotic centrosome proteins and complexes	3.15E-05	3.58E-05
Centrosome maturation	3.15E-05	3.58E-05
CLEC7A (Dectin-1) signaling	3.22E-05	3.62E-05
Autodegradation of Cdh1 by Cdh1:APC/C	3.28E-05	3.64E-05
Vesicle-mediated transport	3.63E-05	3.96E-05
APC/C-mediated degradation of cell cycle proteins	3.71E-05	3.96E-05
Regulation of mitotic cell cycle	3.71E-05	3.96E-05
mRNA Splicing - Major Pathway	3.76E-05	3.96E-05
mRNA Splicing	3.76E-05	3.96E-05
Formation of a pool of free 40S subunits	3.81E-05	3.97E-05
Anchoring of the basal body to the plasma membrane	0.000039	4.02E-05
Cyclin A:Cdk2-associated events at S phase entry	4.02E-05	4.1E-05
Signalling by NGF	4.06E-05	4.1E-05
M/G1 Transition	4.45E-05	4.45E-05
