# Supplementary material for: Modulation of microRNome by Human Cytomegalovirus and Human Herpesvirus 6 Infection in Human Dermal Fibroblasts: Possible Significance in the Induction of Fibrosis in Systemic Sclerosis
Source: Cells. 2021 Apr 29;10(5):1060. doi: 10.3390/cells10051060 (PMC8146000; doi:10.3390/cells10051060)
Supplement: Supplementary file 1 [file cells-10-01060-s001.zip › Table S4.pdf]

Table S4. Enrichment analysis based on differentially expressed miRNAs at early times post HHV-6 infection (0 and 4 d.p.i.)

Pathways	*p* val	adj *p* val
Gene Expression	4.93E-31	4.93E-29
Cell Cycle, Mitotic	1.31E-15	6.55E-14
Infectious disease	1.05E-14	3.5E-13
Cell Cycle	9.7E-14	2.43E-12
Metabolism of proteins	5.4E-13	1.08E-11
Disease	1.36E-11	2.27E-10
HIV Life Cycle	1.95E-11	2.79E-10
HIV Infection	2.34E-11	2.93E-10
Vesicle-mediated transport	3.31E-11	3.68E-10
Membrane Trafficking	4.49E-11	4.49E-10
Late Phase of HIV Life Cycle	1.47E-10	1.34E-09
DNA Repair	1.1E-08	9.17E-08
Transcription	2.24E-08	1.72E-07
Cell Cycle Checkpoints	3.82E-08	2.61E-07
Mitotic G2-G2/M phases	3.92E-08	2.61E-07
G2/M Transition	5.74E-08	3.54E-07
Mitotic Metaphase and Anaphase	6.02E-08	3.54E-07
Metabolism	6.66E-08	3.56E-07
Generic Transcription Pathway	6.99E-08	3.56E-07
Mitotic Anaphase	7.11E-08	3.56E-07
S Phase	1.15E-07	5.48E-07
M Phase	1.57E-07	7.14E-07
Translation	1.72E-07	7.48E-07
Beta-catenin independent WNT signaling	2.39E-07	9.96E-07
Separation of Sister Chromatids	2.66E-07	1.06E-06
DNA Replication	3.16E-07	1.22E-06
Influenza Infection	3.29E-07	1.22E-06
Organelle biogenesis and maintenance	4.29E-07	1.53E-06
Processing of Capped Intron-Containing Pre-mRNA	5.58E-07	1.92E-06
Developmental Biology	7.35E-07	2.45E-06
Influenza Life Cycle	7.92E-07	2.53E-06
Synthesis of DNA	8.08E-07	2.53E-06
Mitotic G1-G1/S phases	9.55E-07	2.89E-06
mRNA Splicing - Major Pathway	1.12E-06	3.2E-06
mRNA Splicing	1.12E-06	3.2E-06
Signalling by NGF	1.52E-06	4.18E-06
C-type lectin receptors (CLRs)	1.59E-06	4.18E-06
Influenza Viral RNA Transcription and Replication	1.59E-06	4.18E-06
Post-translational protein modification	1.76E-06	4.51E-06
GTP hydrolysis and joining of the 60S ribosomal subunit	3.17E-06	7.8E-06
NGF signalling via TRKA from the plasma membrane	3.2E-06	7.8E-06

Regulation of mRNA stability by proteins that bind AU-rich elements	3.59E-06	8.36E-06
L13a-mediated translational silencing of Ceruloplasmin expression	3.76E-06	8.36E-06
3' -UTR-mediated translational regulation	3.76E-06	8.36E-06
RNA Polymerase II Transcription	3.76E-06	8.36E-06
Programmed Cell Death	3.9E-06	8.48E-06
Eukaryotic Translation Initiation	5.1E-06	1.06E-05
Cap-dependent Translation Initiation	5.1E-06	1.06E-05
Signaling by EGFR	6.05E-06	1.22E-05
Apoptosis	6.12E-06	1.22E-05
Signaling by TGF-beta Receptor Complex	6.42E-06	1.26E-05
G1/S Transition	8.84E-06	0.000017
Immune System	9.63E-06	1.79E-05
Platelet activation, signaling and aggregation	9.69E-06	1.79E-05
TRAF6 Mediated Induction of proinflammatory cytokines	0.00001	1.82E-05
VEGFA-VEGFR2 Pathway	1.03E-05	1.84E-05
Regulation of PLK1 Activity at G2/M Transition	1.09E-05	1.91E-05
Cellular responses to stress	1.19E-05	2.05E-05
APC/C-mediated degradation of cell cycle proteins	0.000013	2.17E-05
Regulation of mitotic cell cycle	0.000013	2.17E-05
Nonsense-Mediated Decay (NMD)	1.37E-05	2.21E-05
Nonsense Mediated Decay (NMD) enhanced by the Exon Junction Complex (EJC)	1.37E-05	2.21E-05
Signaling by VEGF	1.47E-05	2.33E-05
SUMOylation	1.57E-05	2.38E-05
M/G1 Transition	1.57E-05	2.38E-05
DNA Replication Pre-Initiation	1.57E-05	2.38E-05
SRP-dependent cotranslational protein targeting to membrane	1.61E-05	2.38E-05
Signaling by PDGF	1.67E-05	2.38E-05
Toll Like Receptor 10 (TLR10) Cascade	1.69E-05	2.38E-05
Toll Like Receptor 5 (TLR5) Cascade	1.69E-05	2.38E-05
MyD88 cascade initiated on plasma membrane	1.69E-05	2.38E-05
CLEC7A (Dectin-1) signaling	1.74E-05	2.42E-05
MyD88-independent TLR3/TLR4 cascade	1.88E-05	2.54E-05
Toll Like Receptor 3 (TLR3) Cascade	1.88E-05	2.54E-05
Transcription of the HIV genome	2.05E-05	2.71E-05
Formation of a pool of free 40S subunits	2.06E-05	2.71E-05
Signaling by Interleukins	2.23E-05	2.86E-05
PCP/CE pathway	2.26E-05	2.86E-05
TRIF-mediated TLR3/TLR4 signaling	2.26E-05	2.86E-05
Diseases of signal transduction	0.000025	3.13E-05
G2/M Checkpoints	2.81E-05	3.43E-05
Nucleotide Excision Repair	2.81E-05	3.43E-05
RNA Polymerase II Pre-transcription Events	3.01E-05	3.63E-05
Regulation of APC/C activators between G1/S and early anaphase	3.25E-05	3.87E-05

Downstream signaling events of B Cell Receptor (BCR)	3.29E-05	3.87E-05
SUMOylation of DNA damage response and repair proteins	3.89E-05	4.47E-05
SUMO E3 ligases SUMOylate target proteins	3.89E-05	4.47E-05
Deadenylation-dependent mRNA decay	4.24E-05	4.76E-05
MAP kinase activation in TLR cascade	4.24E-05	4.76E-05
Nonsense Mediated Decay (NMD) independent of the Exon Junction Complex (EJC)	4.74E-05	5.27E-05
Cyclin E associated events during G1/S transition	5.34E-05	5.87E-05
Signaling by the B Cell Receptor (BCR)	6.31E-05	0.000065
Cytokine Signaling in Immune system	6.38E-05	0.000065
Autodegradation of Cdh1 by Cdh1:APC/C	6.47E-05	0.000065
Downstream signal transduction	0.000065	0.000065
Signaling by FGFR	0.000065	0.000065
