# Supplementary material for: Modulation of microRNome by Human Cytomegalovirus and Human Herpesvirus 6 Infection in Human Dermal Fibroblasts: Possible Significance in the Induction of Fibrosis in Systemic Sclerosis
Source: Cells. 2021 Apr 29;10(5):1060. doi: 10.3390/cells10051060 (PMC8146000; doi:10.3390/cells10051060)
Supplement: Supplementary file 1 [file cells-10-01060-s001.zip › Table S5.pdf]

Table S5. Enrichment analysis based on differentially expressed miRNAs at intermediate time post HHV-6 infection (7 d.p.i.)

Pathways	*p* val	adj *p* val
Gene Expression	1.08E-27	1.08E-25
Metabolism of proteins	1.18E-13	5.9E-12
Cell Cycle, Mitotic	1.03E-12	3.43E-11
Organelle biogenesis and maintenance	5.16E-12	1.29E-10
Infectious disease	2.36E-11	4.72E-10
Cell Cycle	4.32E-11	6.56E-10
Metabolism	4.59E-11	6.56E-10
DNA Repair	2.52E-10	3.15E-09
HIV Life Cycle	5.56E-10	6.18E-09
HIV Infection	8.29E-10	8.29E-09
Developmental Biology	9.64E-10	8.76E-09
Late Phase of HIV Life Cycle	2.71E-09	2.26E-08
Membrane Trafficking	2.97E-09	2.28E-08
Vesicle-mediated transport	1.26E-08	9E-08
Disease	3.15E-08	2.1E-07
Generic Transcription Pathway	4.19E-08	2.62E-07
Transcription	6.24E-08	3.67E-07
Post-translational protein modification	1.21E-07	6.72E-07
Assembly of the primary cilium	2.21E-07	1.16E-06
Translation	3.32E-07	1.66E-06
Mitotic G2-G2/M phases	5.78E-07	2.75E-06
Mitotic G1-G1/S phases	6.87E-07	3.12E-06
Axon guidance	7.43E-07	3.16E-06
G2/M Transition	7.79E-07	3.16E-06
Mitochondrial translation	7.91E-07	3.16E-06
Cell Cycle Checkpoints	1.05E-06	4.04E-06
Mitotic Metaphase and Anaphase	1.43E-06	5.3E-06
Mitotic Anaphase	1.63E-06	5.82E-06
Processing of Capped Intron-Containing Pre-mRNA	1.86E-06	6.34E-06
Mitochondrial translation initiation	2.03E-06	6.34E-06
Mitochondrial translation elongation	2.03E-06	6.34E-06
Mitochondrial translation termination	2.03E-06	6.34E-06
S Phase	2.46E-06	7.45E-06
mRNA Splicing - Major Pathway	3.27E-06	9.34E-06
mRNA Splicing	3.27E-06	9.34E-06
Programmed Cell Death	3.97E-06	1.1E-05
Beta-catenin independent WNT signaling	4.33E-06	1.16E-05
SUMOylation	4.46E-06	1.16E-05
Separation of Sister Chromatids	4.51E-06	1.16E-05
MyD88-independent TLR3/TLR4 cascade	5.21E-06	1.27E-05

Toll Like Receptor 3 (TLR3) Cascade	5.21E-06	1.27E-05
Apoptosis	5.8E-06	1.38E-05
TRIF-mediated TLR3/TLR4 signaling	6.1E-06	1.42E-05
Respiratory electron transport	7.13E-06	1.62E-05
Activated TLR4 signalling	8.31E-06	1.85E-05
RNA Polymerase II Transcription	8.73E-06	1.86E-05
Influenza Infection	8.74E-06	1.86E-05
SUMOylation of DNA damage response and repair proteins	9.76E-06	1.99E-05
SUMO E3 ligases SUMOylate target proteins	9.76E-06	1.99E-05
M Phase	0.000011	0.000022
Signaling by TGF-beta Receptor Complex	1.34E-05	2.63E-05
Signalling by NGF	1.42E-05	2.73E-05
Influenza Life Cycle	0.000017	3.15E-05
Asparagine N-linked glycosylation	0.000017	3.15E-05
G1/S Transition	1.75E-05	3.18E-05
Eukaryotic Translation Initiation	1.95E-05	3.42E-05
Cap-dependent Translation Initiation	1.95E-05	3.42E-05
DNA Replication	2.66E-05	4.59E-05
Cellular responses to stress	2.83E-05	4.63E-05
Influenza Viral RNA Transcription and Replication	2.89E-05	4.63E-05
Toll Like Receptor 10 (TLR10) Cascade	2.92E-05	4.63E-05
Toll Like Receptor 5 (TLR5) Cascade	2.92E-05	4.63E-05
MyD88 cascade initiated on plasma membrane	2.92E-05	4.63E-05
CLEC7A (Dectin-1) signaling	3.05E-05	4.77E-05
Signaling by Wnt	3.13E-05	4.82E-05
ISG15 antiviral mechanism	3.42E-05	5.03E-05
Antiviral mechanism by IFN-stimulated genes	3.42E-05	5.03E-05
Transcription of the HIV genome	3.42E-05	5.03E-05
Fatty acid, triacylglycerol, and ketone body metabolism	4.14E-05	5.91E-05
C-type lectin receptors (CLRs)	4.14E-05	5.91E-05
Toll Like Receptor 4 (TLR4) Cascade	4.61E-05	6.49E-05
RNA Polymerase II Pre-transcription Events	4.68E-05	0.000065
Regulation of PLK1 Activity at G2/M Transition	4.81E-05	6.53E-05
SRP-dependent cotranslational protein targeting to membrane	0.000049	6.53E-05
GTP hydrolysis and joining of the 60S ribosomal subunit	0.000049	6.53E-05
Synthesis of DNA	5.29E-05	6.96E-05
L13a-mediated translational silencing of Ceruloplasmin expression	5.59E-05	7.17E-05
3' -UTR-mediated translational regulation	5.59E-05	7.17E-05
Signaling by Interleukins	6.36E-05	8.05E-05
Cyclin E associated events during G1/S transition	7.48E-05	9.35E-05
VEGFA-VEGFR2 Pathway	7.97E-05	9.84E-05
Autodegradation of Cdh1 by Cdh1:APC/C	8.75E-05	0.000107
Cyclin A:Cdk2-associated events at S phase entry	0.000102	0.000123

Anchoring of the basal body to the plasma membrane	0.00012	0.000143
Host Interactions of HIV factors	0.000125	0.000147
MyD88:Mal cascade initiated on plasma membrane	0.000132	0.000148
Toll Like Receptor TLR1:TLR2 Cascade	0.000132	0.000148
Toll Like Receptor TLR6:TLR2 Cascade	0.000132	0.000148
Toll Like Receptor 2 (TLR2) Cascade	0.000132	0.000148
Transcriptional regulation of white adipocyte differentiation	0.00014	0.000156
NGF signalling via TRKA from the plasma membrane	0.000149	0.000162
Platelet activation, signaling and aggregation	0.000149	0.000162
Signaling by EGFR	0.000154	0.000165
Regulation of mRNA stability by proteins that bind AU-rich elements	0.000157	0.000165
Signaling by VEGF	0.000158	0.000165
Mitotic Prometaphase	0.000158	0.000165
TRAF6 Mediated Induction of proinflammatory cytokines	0.000164	0.000169
Cellular response to heat stress	0.000177	0.000181
Signaling by NOTCH	0.00018	0.000182
Toll-Like Receptors Cascades	0.000185	0.000185
