# Supplementary material for: Modulation of microRNome by Human Cytomegalovirus and Human Herpesvirus 6 Infection in Human Dermal Fibroblasts: Possible Significance in the Induction of Fibrosis in Systemic Sclerosis
Source: Cells. 2021 Apr 29;10(5):1060. doi: 10.3390/cells10051060 (PMC8146000; doi:10.3390/cells10051060)
Supplement: Supplementary file 1 [file cells-10-01060-s001.zip › Table S6.pdf]

Table S6. Enrichment analysis based on differentially expressed miRNAs at late times post HHV-6 infection (10 and 14 d.p.i.)

**Pathways**	***p* val**	**adj *p* val**
Gene Expression	4E-20	4E-18
Metabolism of proteins	2.18E-13	1.09E-11
Cell Cycle, Mitotic	4.01E-11	1.34E-09
Signalling by NGF	1.36E-10	2.74E-09
Infectious disease	1.82E-10	2.74E-09
HIV Infection	1.85E-10	2.74E-09
Membrane Trafficking	1.92E-10	2.74E-09
Disease	2.73E-10	3.41E-09
HIV Life Cycle	1.36E-09	1.44E-08
Cell Cycle	1.44E-09	1.44E-08
Post-translational protein modification	3.21E-09	2.92E-08
DNA Repair	4.42E-09	3.68E-08
Generic Transcription Pathway	5.45E-09	4.19E-08
Late Phase of HIV Life Cycle	9.48E-09	6.77E-08
Diseases of signal transduction	1.42E-08	9.47E-08
NGF signalling via TRKA from the plasma membrane	2.45E-08	1.53E-07
Signaling by EGFR	4.07E-08	2.39E-07
Signaling by the B Cell Receptor (BCR)	8.5E-08	4.72E-07
Mitotic G2-G2/M phases	1.67E-07	8.79E-07
Vesicle-mediated transport	2.2E-07	1.1E-06
G2/M Transition	2.43E-07	1.16E-06
Immune System	3.61E-07	1.64E-06
Asparagine N-linked glycosylation	3.81E-07	1.66E-06
Mitotic G1-G1/S phases	4.46E-07	1.86E-06
Signaling by ERBB2	5.87E-07	2.35E-06
Signaling by PDGF	6.54E-07	2.45E-06
Developmental Biology	6.62E-07	2.45E-06
Downstream signaling events of B Cell Receptor (BCR)	1.34E-06	4.79E-06
Metabolism	1.72E-06	5.93E-06
S Phase	2.16E-06	7.2E-06
Downstream signal transduction	2.75E-06	7.64E-06
Signaling by FGFR	2.75E-06	7.64E-06
Signaling by FGFR1	2.75E-06	7.64E-06
Signaling by FGFR2	2.75E-06	7.64E-06
Signaling by FGFR3	2.75E-06	7.64E-06
Signaling by FGFR4	2.75E-06	7.64E-06
Transcription	3.39E-06	9.16E-06
EPH-Ephrin signaling	3.58E-06	9.42E-06
Signaling by TGF-beta Receptor Complex	3.88E-06	9.95E-06
Organelle biogenesis and maintenance	5.02E-06	1.26E-05
VEGFA-VEGFR2 Pathway	5.78E-06	1.41E-05

Translation	0.000006	1.41E-05
DAP12 signaling	6.24E-06	1.41E-05
Programmed Cell Death	6.24E-06	1.41E-05
Regulation of PLK1 Activity at G2/M Transition	6.36E-06	1.41E-05
Processing of Capped Intron-Containing Pre-mRNA	7.29E-06	1.56E-05
Fc epsilon receptor (FCERI) signaling	7.33E-06	1.56E-05
Signaling by VEGF	8.04E-06	1.68E-05
Axon guidance	9.05E-06	1.85E-05
Apoptosis	9.81E-06	1.96E-05
Adaptive Immune System	1.16E-05	2.27E-05
Signaling by SCF-KIT	1.23E-05	2.37E-05
mRNA Splicing - Major Pathway	1.41E-05	2.61E-05
mRNA Splicing	1.41E-05	2.61E-05
Biosynthesis of the N-glycan precursor (dolichol lipid-linked oligosaccharide, LLO) and transfer to a nascent protein	1.58E-05	2.87E-05
Signaling by NOTCH	1.73E-05	2.92E-05
Downstream signaling of activated FGFR1	1.75E-05	2.92E-05
Downstream signaling of activated FGFR2	1.75E-05	2.92E-05
Downstream signaling of activated FGFR3	1.75E-05	2.92E-05
Downstream signaling of activated FGFR4	1.75E-05	2.92E-05
Nucleotide Excision Repair	1.91E-05	3.13E-05
G1/S Transition	2.36E-05	3.64E-05
MyD88:Mal cascade initiated on plasma membrane	0.000024	3.64E-05
Toll Like Receptor TLR1:TLR2 Cascade	0.000024	3.64E-05
Toll Like Receptor TLR6:TLR2 Cascade	0.000024	3.64E-05
Toll Like Receptor 2 (TLR2) Cascade	0.000024	3.64E-05
Platelet activation, signaling and aggregation	3.15E-05	4.7E-05
Cellular response to heat stress	0.000035	5.15E-05
DNA Replication	0.000039	5.65E-05
PI3K/AKT Signaling in Cancer	4.25E-05	6.07E-05
RNA Polymerase II Transcription	0.000044	6.2E-05
Assembly of the primary cilium	5.07E-05	7.04E-05
GAB1 signalosome	5.44E-05	7.45E-05
Transcription-coupled NER (TC-NER)	5.58E-05	7.54E-05
SUMOylation	6.07E-05	8.09E-05
PI3K/AKT activation	6.41E-05	8.43E-05
HIV Transcription Initiation	6.91E-05	8.43E-05
RNA Polymerase II HIV Promoter Escape	6.91E-05	8.43E-05
RNA Polymerase II Promoter Escape	6.91E-05	8.43E-05
RNA Polymerase II Transcription Pre-Initiation And Promoter Opening	6.91E-05	8.43E-05
RNA Polymerase II Transcription Initiation	6.91E-05	8.43E-05
RNA Polymerase II Transcription Initiation And Promoter Clearance	6.91E-05	8.43E-05
Beta-catenin independent WNT signaling	7.55E-05	9.1E-05
TRIF-mediated TLR3/TLR4 signaling	8.66E-05	0.000103

Synthesis of DNA	0.000089	0.000105
TRAF6 Mediated Induction of proinflammatory cytokines	9.44E-05	0.00011
Signaling by ERBB4	9.76E-05	0.000111
PI3K events in ERBB4 signaling	0.000105	0.000111
PIP3 activates AKT signaling	0.000105	0.000111
PI3K events in ERBB2 signaling	0.000105	0.000111
PI-3K cascade:FGFR	0.000105	0.000111
Transcriptional activity of SMAD2/SMAD3:SMAD4 heterotrimer	0.000106	0.000111
Transcription of the HIV genome	0.000107	0.000111
Activated TLR4 signalling	0.000123	0.000127
SUMOylation of DNA damage response and repair proteins	0.000147	0.000148
SUMO E3 ligases SUMOylate target proteins	0.000147	0.000148
Mitotic Metaphase and Anaphase	0.000155	0.000155
