# Supplementary figures and images for: Modulation of microRNome by Human Cytomegalovirus and Human Herpesvirus 6 Infection in Human Dermal Fibroblasts: Possible Significance in the Induction of Fibrosis in Systemic Sclerosis
Source: Cells. 2021 Apr 29;10(5):1060. doi: 10.3390/cells10051060 (PMC8146000; doi:10.3390/cells10051060)

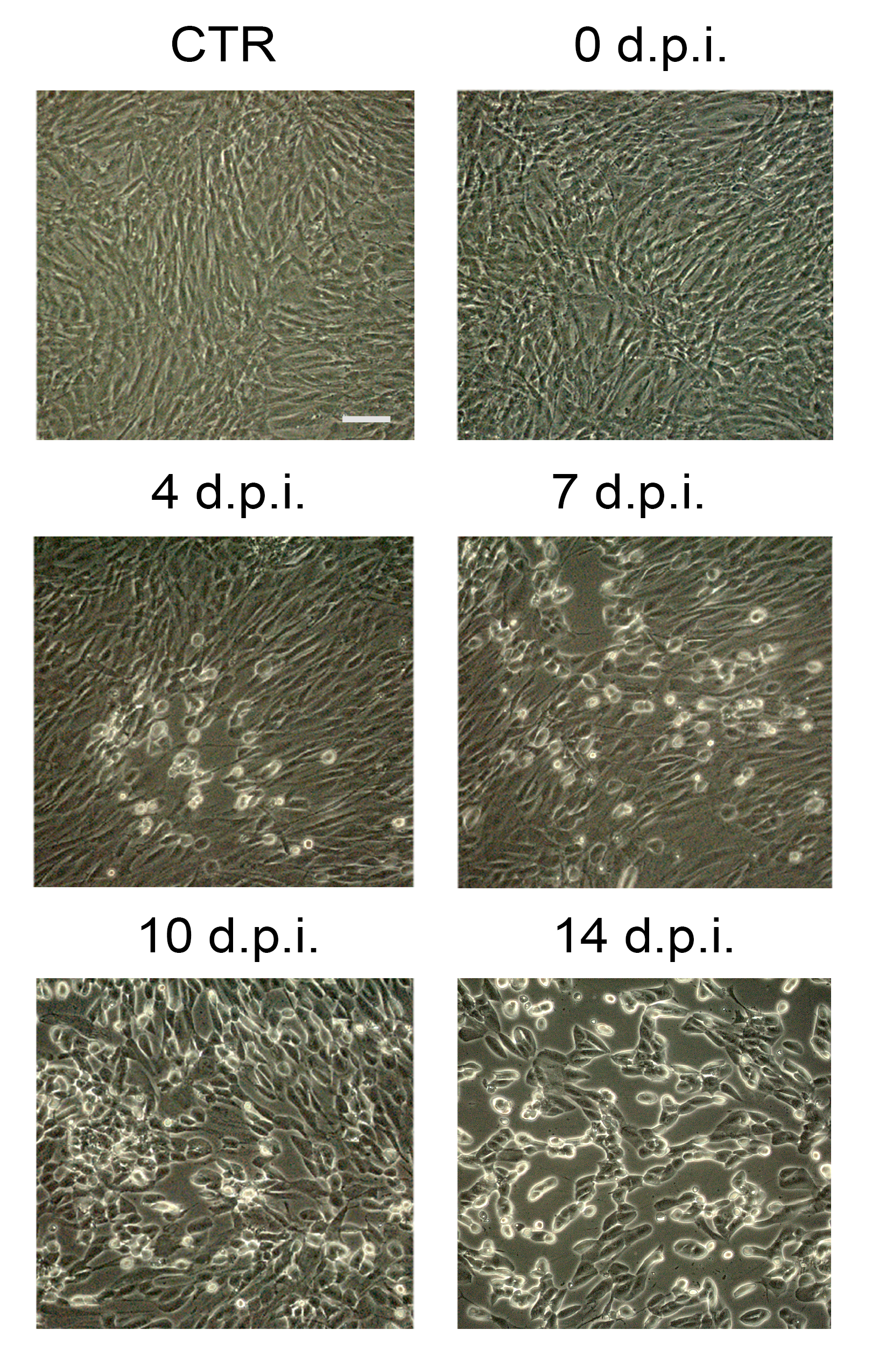

Supplement: Supplementary file 1 [file cells-10-01060-s001.zip › Figure S1.tif]
